# *Helicobacter pylori* γ-glutamyltransferase is linked to proteomic adaptions important for colonization

**DOI:** 10.1080/19490976.2025.2488048

**Published:** 2025-04-09

**Authors:** Sonja Fuchs, Michaela K. Fiedler, Nicole Heiduk, Andreas Wanisch, Cora Mibus, Dharmesh Singh, Aleksandra W. Debowski, Barry J. Marshall, Michael Vieth, Christine Josenhans, Sebastian Suerbaum, Stephan A. Sieber, Markus Gerhard, Raquel Mejías-Luque

**Affiliations:** a Institute for Medical Microbiology, Immunology and Hygiene, Department of Preclinical Medicine, TUM School of Medicine and Health, Technical University of Munich (TUM), Munich, Germany; b Center for Functional Protein Assemblies (CPA), Chair of Organic Chemistry II, Department Biosciences, TUM School of Natural Sciences, Technical University of Munich (TUM), Garching, Germany; c Marshall Centre for Infectious Disease Research and Training, School of Biomedical Sciences, The University of Western Australia, Nedlands, Australia; d School of Molecular Sciences, The University of Western Australia, Crawley, Australia; e Institute of Pathology, Friedrich-Alexander-University Erlangen-Nuremberg, Klinikum Bayreuth, Bayreuth, Germany; f Max von Pettenkofer Institute, Faculty of Medicine, Medical Microbiology and Hospital Epidemiology, Ludwig-Maximilians-Universität (LMU) Munich, Munich, Germany; g DZIF - German Center for Infection Research, Partner Site Munich, Munich, Germany

**Keywords:** Gamma-glutamyltransferase, *Helicobacter pylori*, colonization, acid resistance, bacterial metabolism

## Abstract

*Helicobacter pylori* γ-glutamyltransferase (gGT) is a virulence factor that promotes bacterial colonization and immune tolerance. Although some studies addressed potential functional mechanisms, the supportive role of gGT for *in vivo* colonization remains unclear. Additionally, it is unknown how different gGT expression levels may lead to compensatory mechanisms ensuring infection and persistence. Hence, it is crucial to unravel the *in vivo* function of gGT. We assessed acid survival under conditions mimicking the human gastric fluid and elevated the pH in the murine stomach prior to *H. pylori* infection to link gGT-mediated acid resistance to colonization. By comparing proteomes of gGT-proficient and -deficient isolates before and after infecting mice, we investigated proteomic adaptations of gGT-deficient bacteria during infection. Our data indicate that gGT is crucial to sustain urease activity in acidic environments, thereby supporting survival and successful colonization. Absence of gGT triggers expression of proteins involved in the nitrogen and iron metabolism and boosts the expression of adhesins and flagellar proteins during infection, resulting in increased motility and adhesion capacity. In summary, gGT-dependent mechanisms confer a growth advantage to the bacterium in the gastric environment, which renders gGT a valuable target for the development of new treatments against *H. pylori* infection.

## Introduction

*Helicobacter pylori* inhabits the stomach of more than 40% of the world’s population with varying prevalence across different regions.^1^ Although most patients are asymptomatic, *H. pylori* causes gastric inflammation, elevating the risk for severe gastroduodenal diseases including ulcers, gastric cancer, or MALT lymphoma.^1^ The severity of *H. pylori*-induced diseases is attributed to an array of virulence factors, including γ-glutamyl transferase (gGT) that was shown to contribute to gastric cancer development^2^ and peptic ulcer disease.^3^

*H. pylori* gGT catalyzes the hydrolysis of glutamine and glutathione, thereby releasing the metabolites glutamate, ammonia and cysteinyl glycine (Cys-Gly) into the bacterial microenvironment.^4^ It is well established that gGT-dependent alterations in metabolite concentrations affect host cells. Elevated ammonia levels exert cytotoxic effects on gastric epithelial cells,^5,6^ while the release of reactive oxygen species resulting from gGT activity triggers the secretion of pro-inflammatory interleukin 8 (IL-8) and the induction of apoptosis in gastric epithelial cells.^3^ In addition to its effect on gastric epithelial cells, gGT activity also facilitates immune evasion of the bacterium.^7^ *H. pylori* gGT-induced glutamine deprivation hampers proliferation of T cells^8^ and their effector function.^9^ Simultaneously, a gGT-mediated increase in glutamate levels skews dendritic cells toward a more tolerogenic phenotype, favoring the proliferation of regulatory T cells that subsequently suppress effector T cells.^10^

Beyond its impact on host cells, *H. pylori* gGT also promotes bacterial colonization, as evidenced by several studies demonstrating that *ggt* deletion diminishes colonization in murine infection models.^5,8,11,12^ However, even though *H. pylori* gGT is recognized as a key colonization factor, a small fraction of gGT-deficient bacteria is able to colonize and persist in the stomach at similar levels compared to the wild type strain (wt), indicating that the gGT-deficient *H. pylori* strain is somehow able to compensate for the loss of gGT activity.^8^ However, the underlying mechanisms that allow gGT-deficient bacteria to adapt in the absence of the enzyme, and to provide an advantage to the bacterium during colonization, remain elusive. Nevertheless, the fact that all gastric but not all enterohepatic *Helicobacter* express gGT suggests that the enzyme plays a central role specifically in the stomach environment.^13^

A differential immune cell infiltration triggered by the gGT enzyme was proposed as a possible explanation for the initial colonization hurdle observed for gGT-deficient strains. However, this hypothesis was dismissed because a reduced colonization capacity of gGT-deficient bacteria was also observed in the absence of immune cells.^8^ *In vitro* studies further suggested that gGT might be metabolically important for the bacterium, providing another plausible explanation for the supporting effect of gGT during colonization. In this context, *H. pylori* gGT was shown to enhance bacterial acid survival *in vitro* in the presence of urea^14^ and to contribute to *H. pylori*’s carbon and nitrogen metabolism by enabling the bacterium to use extracellular glutamine. *H. pylori* is unable to take up glutamine directly but has to hydrolyze the amino acid to glutamate first, which is then taken up via the sole glutamate transporter GltS, and subsequently incorporated into metabolic pathways.^4,13^ The inability of *gltS*-deficient *H. pylori* to infect Mongolian gerbils stresses that this gGT-linked uptake system is also relevant *in vivo*. Apart from glutamine, gGT-derived Cys-Gly might also support *H. pylori*’s metabolism as this metabolite has recently been shown to be internalized by the bacterium in a gGT-dependent manner^15^ and has already been found to be essential for the proliferation of other pathogens during infection including *Francisella tularensis*^16^ and *Neisseria meningitidis*.^17^

Considering that different enzymatic activity levels have been observed in clinical isolates and that gGT activity has a high impact on bacterial growth and colonization, it is essential to identify compensatory mechanisms triggered in the absence of gGT, and to understand the metabolic role of this virulence factor in more detail. To this end, we have analyzed changes in bacterial protein expression occurring during infection of mice with wt or gGT-deficient *H. pylori* and linked the contribution of gGT to acid resistance to colonization. Our findings indicate that gGT favors acid survival by supporting the key acid survival factor urease and has essential roles in different metabolic pathways important for the growth and infection capacity of *H. pylori*.

## Materials and methods

### *H. pylori* strains and culture conditions

*H. pylori* was grown in microaerobic conditions at 37°C, 10% (*v*/*v*) CO_2_ and 5% (*v*/*v*) O_2_ on WC sheep blood agar plates supplemented with Dent (Oxoid) or in BHI medium supplemented with 10% FCS shaking at 120 rpm. In this study, the *H. pylori* laboratory strains G27,^18^ and PMSS1^19^ as well as clinical isolates were used. PMSS1 ∆*ureAB* was generated by transformation of genomic DNA from G27 ∆*ureAB*.^20^ Mutant strains were grown with antibiotic selection in the growth medium (50 µg/ml kanamycin, 10 µg/ml streptomycin or 10 µg/ml chloramphenicol). All strains used in this study are listed in Table S1. To induce gGT activity in *H. pylori g:O1 t*, 100 ng/ml anhydrotetracycline (ATc) was added to the culture for 24 h, if not stated otherwise. If *H. pylori g:O1 t* was used for experiments, gGT activity was induced in the pre-culture and during the experiment except as otherwise indicated. In some experiments *H. pylori* was grown in acidified Brucella medium (pH 5) supplemented with 20% FCS. The pH was adjusted with hydrogen chloride and pH was monitored during the experiment with a portable pH measuring device (FiveGo pH meter F2-Std-Kit, Mettler-Toledo).

### Mouse experiments

Female C57BL/6 wild-type mice (Envigo) were housed under specific pathogen-free conditions and were fed *ad libitum*. Six- to eight-week-old mice were infected with *H. pylori* PMSS1 wt or with PMSS1 ∆*ggt* by oral gavage with 2 × 10^8^ bacteria resuspended in 200 µl BHI with 20% FCS. Mice were challenged twice with *H. pylori* at a time interval of two days. A group of mice received 200 µl sodium bicarbonate (10% w/v) 10 min before infection to elevate the pH in the murine stomach. Colonization was analyzed by plating serial dilutions of a longitudinal piece of stomach homogenized in BHI/20% FCS on WC-dent blood agar plates supplemented with bacitracin (200 μg/ml), nalidixic acid (10 μg/ml), and polymyxin B (3 μg/ml). After 5 days of culture, single clones were counted and expanded on WC-dent plates for 2 days and then either frozen for later use or directly grown in liquid culture for proteomic analysis. To compare the protein expression before infection with the proteome after infection, a control sample was prepared: The aliquot initially used for mouse infections was taken into culture on the same day when mice were sacrificed and was expanded and grown in liquid culture using the same time schedule as for the re-isolates. All experiments were approved by the Bavarian Government (Regierung von Oberbayern, ROB-55.2-2532.Vet_02-19-95) and conducted in compliance with European guidelines for the care and use of laboratory animals.

### Proteomics

*H. pylori* strains were grown to the early stationary growth phase or for a maximum of 35 h, washed twice with PBS, and lysed in RIPA buffer (50 mm Tris, 150 mm NaCl, 1 mm EGTA, 1% Igepal, 0.25% sodium deoxycholate, pH 7.4) using ultrasonication. The insoluble fraction was separated by centrifugation (30 min, 10 000 g, 4 °C) and protein concentration in the supernatant was determined by a commercially available BCA assay (ThermoFisher Scientific). The same protein amount was used for each measurement.

Proteins were precipitated with ice-cold acetone (−20°C, overnight), centrifuged (21 000 g, 15 min, 4 °C) and dissolved in cold methanol (−80 °C) using sonication (10 s, 10%, 5× cycle). Proteins were resuspended in denaturation buffer (7 M urea, 2 M thiourea in 20 mm HEPES, pH 7.5) and reduced in the presence of 10 mm TCEP (1 h, 37°C, 600 rpm). Alkylation was performed with 10 mm IAA (30 min, 1 h, 600 rpm) and was quenched by adding DTT to a final concentration of 10 mm (30 min, 600 rpm). Proteins were pre-digested with LysC (Promega) for 2 h at 25°C and digested with trypsin (Promega, 37°C, overnight) in TEAB buffer (50 mm, triethylammonium bicarbonate). Digestion was stopped by acidification with formic acid to a pH below 3. SepPak C18 cartridges (Waters) were used for desalting. Cartridges were equilibrated with 1 ml elution buffer (80% acetonitrile, 0.5% formic acid) and 0.1% TFA, before peptides were loaded on the column. Washing was done with 0.1% TFA (2× 1 ml) and with 0.5% formic acid (1× 0.5 ml). Peptides were eluted with elution buffer (1× 500 μl, 1 × 250 μl) and dried by lyophilization. After reconstitution in 1% formic acid to a final concentration of 1 µg/µl, peptides were filtered through centrifugal filter units (Merck, PVDF, 0.22 µm) before being transferred to MS vials.

MS analysis was performed on an Orbitrap Fusion mass spectrometer coupled to Ultimate3000 nano-HPLC via a *Nanospray Flex* Ion Source (ThermoFisher Scientific). Samples were loaded on the trap column (AcclaimPepMap 100 C18 (75 μm × 2 cm) trap/flow rate: 5 µl/min, 0.1% TFA) and separated on the Aurora Ultimate^TM^ columns (2^nd^ generation, 75 µm × 25 cm, *ionopticks*). Both columns were constantly heated to 40°C. For separation, a buffer B gradient (0.1% formic acid in acetonitrile) with a flow rate of 400 nL/min was applied (5–22% buffer B for 112 min, to 32% buffer B in 10 min, to 90% buffer B in 10 min, hold for 10 min, to 5% buffer B in 0.1 min, hold 5% buffer B for 9.9 min). The Orbitrap was operated in a cycle time (3 s) data dependent mode. An AGC target of 2e5, a maximum injection time of 50 ms, 60% RF lens and a resolution of 120 000 in a scan range of 300–1500 *m/z* in profile mode was used. Monoisotopic precursor selection and dynamic exclusion (60 s) was turned on. For fragmentation, most intense precursors with charges of 2–7 and intensities greater than 5e3 were chosen. Quadrupole isolation was performed using a range of 1.6 *m/z*. Precursor ions were separated using an AGC target of 1e4 and a maximum injection time of 35 ms. Fragmentation was performed using higher-energy collisional dissociation with a collision energy of 30%. Fragments were detected in the ion trap operating at a rapid scan rate.

Data acquisition was done using Xcalibur software followed by data processing using MaxQuant 1.6.2.10.^21^ Proteins were searched against the data available for *H. pylori* PMSS1 (UP000289502) or G27 (UP000001735) in the UniProt database. Further bioinformatical analysis was performed with Perseus version 1.6.14.10.^22^ Potential contaminants and reverse hits were removed. Label-free quantification intensities were log_2_-transformed and proteins with less than three valid values were filtered off. Missing values were replaced by imputed numbers from a matrix (width 0.3, downshift 1.8, total matrix). To identify relevant differences Student’s t-test was used. Hits with a *p*-value < 0.05 and a Student’s t-test difference of at least |1| were considered to be significant. Proteomics data can be accessed via the PRIDE repository with the identifier PXD050334.

### Urease activity assay

Urease activity was determined as previously described with some modifications.^23^ *H. pylori* (4 × 10^8^ bacteria/ml) was harvested in PEB (100 mm sodium phosphate buffer, pH 7.3, 10 mm EDTA), washed and lysed by 10 min sonication. Lysates were cleared from cell debris by centrifugation. To assess urease activity following exposure to an acidic environment, bacteria were resuspended in McIlvaine buffer (pH 5 or pH 7) and incubated for 2 hours prior to lysis. For measuring enzymatic activity in the supernatant, bacterial supernatants were collected after 2 hours of incubation in McIlvaine buffer. Protein concentrations were determined by a commercially available BCA assay (ThermoScientific). Samples (90 µl) were mixed with 100 µl of reaction buffer (50 mm urea in PEB). The hydrolysis of urea was stopped after 10 min at room temperature by the addition of 100 µl phenol-nitroprusside (Sigma). For color development, 50 µl alkaline hypochlorite (Sigma) was added, and absorption was measured after 1 h (Tecan plate reader, 670 nm). The amount of ammonia released was determined from a standard curve with ammonium chloride (0–500 µM). One unit (U) of enzymatic activity was defined as the amount of enzyme that hydrolyses one µmol of substrate per minute under the conditions of the assay.

### gGT activity assay

gGT activity was determined by a colorimetric assay based on the release of p-nitroaniline from the substrate gGpNA (Sigma). The method was adapted from Meister et al.^24^ and from Schmees et al.^25^ with some modifications. *H. pylori* was resuspended in PBS (2 × 10^8^ bacteria/ml) and incubated for 2 h in microaerobic conditions shaking at 120 rpm. Supernatant and bacteria were separated by centrifugation (5000 *g*, 5 min) and 50 µl of the supernatant was used for the assay. Supernatant and 150 µl reaction buffer (5 mm gGpNA, 100 mm Gly-Gly in 0.1 M Tris buffer, pH 8) were mixed. After 1 h of incubation at 37°C the release of p-nitroaniline was measured (Tecan plate reader, 405 nm). One unit (U) of activity was defined as the amount of enzyme that releases one µmol of p-nitroanilide per minute under the conditions of the assay.

### Iron detection assay

To measure whole iron uptake by *H. pylori*, bacteria (4 × 10^8^ bacteria/ml) were incubated with varying concentrations of iron(III)citrate in PBS, in the presence or absence of GSH or Cys-Gly, for 4 h at 37°C in microaerobic conditions. Iron sources remaining in the supernatant after incubation were quantified using the iron detection reagent developed by Flores et al.^26^ Briefly, sample (50 µl), water (100 µl) and 50 µl detection reagent (6.5 mm ferrozine, 2.5 M ammonium acetate, 1 M ascorbic acid, 2 mm thiourea) were mixed. Absorption was measured after 60 min at 25°C (Tecan plate reader, 562 nm). To measure Fe^2+^ concentrations, the detection solution was prepared without ascorbic acid. Iron concentrations were calculated by comparison to a FeCl_2_ or Fe(III)citrate standard curve (0–500 µM).

### Growth in iron-restricted medium

0.2 × 10^8^ bacteria/ml were resuspended in BHI supplemented with 10% FCS and with deferoxamine mesylate (DFO) at different concentrations (0 µM, 20 µM, 50 µM or 75 µM). Bacteria were grown for 48 h. Growth was measured spectrophotometrically at 600 nm using a BioPhotometer (Eppendorf). Spectrometric growth measurements were normalized to bacterial growth without DFO.

### Acid survival assay

Acid survival was measured by the method described by Miller and Maier with some modifications.^14^ *H. pylori* was grown in liquid culture for 24 h, harvested and washed with PBS once. Bacteria were resuspended in 20 ml of acid challenge buffer at a concentration of 0.2 × 10^8^ bacteria/ml. McIlvaine buffer (pH 3 or pH 5) or fasted state simulated gastric fluid (FaSSGF,^27^ were used. For some experiments acid challenge buffers were supplemented with 8 mm urea. Bacteria were incubated in microaerobic conditions at 37°C shaking at 120 rpm. Survival was measured by plating serial dilutions on WC-dent blood agar plates.

### Soft agar motility assay

*H. pylori* motility was assessed as previously described.^28^ The soft agar plates consisted of Brucella broth, 0.35% (w/v) agar, 10% (v/v) FCS and *H. pylori* selective supplement (Dent). Using a sterile toothpick, *H. pylori* was inoculated into the soft agar. Migration of the bacteria was monitored for up to five days by measuring the diameter of the bacterial halo around the starting point.

### Flow cytometry–based binding assay

AGS cells (ATCC CRL-1739) were cultured in DMEM (Gibco) containing 10% FCS and 1% Pen-Strep (Gibco, 5000 U/ml) at 5% CO_2_ at 37°C. A *H. pylori* culture was adjusted to a concentration of 2 × 10^8^ bacteria/ml in BHI supplemented with 20% FCS and was stained with TAMRA (Sigma, 1 µl TAMRA/ml) for 30 min shaking at 120 rpm in microaerobic conditions at 37°C. AGS cells were detached with trypsin (Gibco), resuspended in antibiotic-free DMEM and stained with DAPI (Sigma, 3 µl DAPI/ml) for 30 min at 37°C. Bacteria and cells were washed twice with PBS to remove excessive dye. AGS cells were seeded in a 96-Well plate (1 × 10^5^ cells/well) in antibiotic-free DMEM supplemented with 10% BHI/20%FCS, mixed with bacteria at a MOI of 5 and medium was topped up to 200 µl. After 30 min at 37°C, unbound bacteria were washed off with PBS in three washing steps. Cells were fixed with 1% PFA in PBS, washed with PBS and resuspended in FACS buffer (0.5% BSA in PBS). Binding was analyzed by flow cytometry (Cytoflex S, Beckman Coulter) by comparing the TAMRA-signals (PE-channel) of AGS cells.

### Genome sequencing and comparison

Genome sequencing was performed using Illumina technology on a MiSeq instrument as previously described.^29^ Genome reconstructions for the *H. pylori* isolates were performed using the Kbase narrative portal.^30^ Paired-end FASTQ files in compressed fastq.gz format were imported into the KBase staging area, decompressed, and uploaded as “Fastq Reads NonInterleaved”. Forward (R1) and reverse (R2) reads for each sample were paired to create a paired-end read library. Individual assemblies were performed using SPAdes (v3.15.3), followed by annotation with Prokka (v1.14.5). The annotated outputs, including genomic sequences and associated features, were exported in GenBank (.gbk) format for downstream analyses. Mauve whole genome aligner^31^ embedded into Geneious Prime 2025.0.2^32^ was used for whole-genome comparison of isolates initially used for mouse infection with re-isolates and the PMSS1 reference genome (GenBank accession number: CP018823).

### Statistical analysis

Results of *in vitro* experiments are presented as mean ± standard deviation (SD) of three independent experiments, if not stated otherwise. Results from *in vivo* experiments are presented as dot plots with medians. The normal distribution of data was tested using the Shapiro-Wilk test. Normally distributed data was analyzed using a t-test or one-way ANOVA followed by multiple Bonferroni corrected comparisons. For non-normally distributed data, Mann-Whitney U-Test was used. Statistical significance was established when *p* < 0.05, while highly statistically significant results had a p-value of *p* < 0.01.

## Results

### Generation of gGT-inducible *H. pylori* strains

The analysis of the function of the virulence factor gGT has mainly relied on comparing *H. pylori* wild type strains (wt) with gGT-deficient deletion mutants (∆*ggt*). However, this approach does not accurately reflect human infections, in which all clinical isolates were found to express gGT,^13^ albeit at varying levels, and the activity of the enzyme determines pathology.^2,3^ Therefore, a model allowing for regulation of the expression levels of gGT was established to study the virulence factor gGT in more depth.

**Figure 1.**
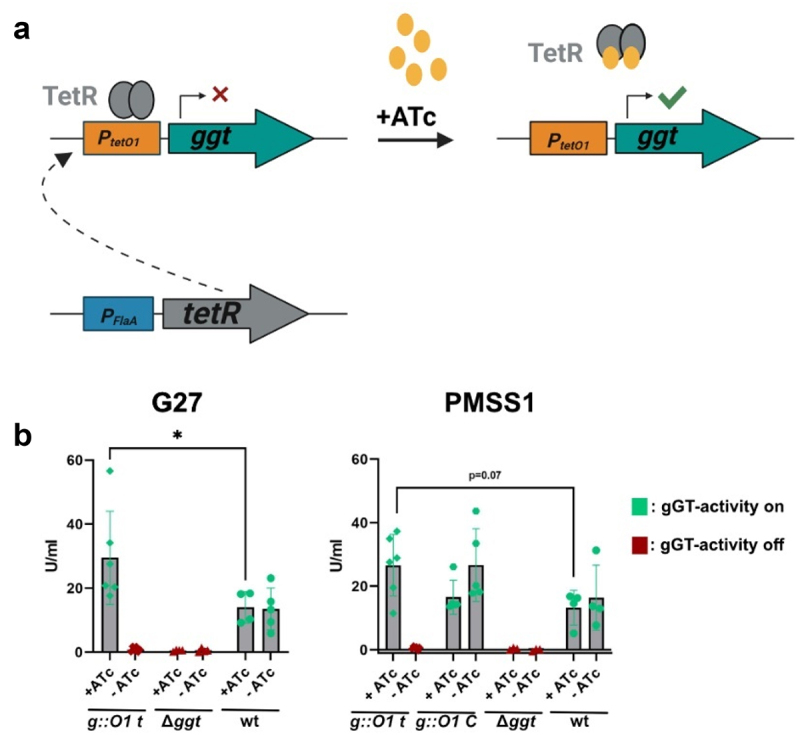
Establishment and characterization of inducible tet-on gGT *H. pylori* strains (a) Graphic representation of the *tet*-on gGT system. The *ggt* promoter was replaced by the *tet* inducible promoter *tetO1* (P_tetO1_). TetR was introduced in the *H. pylori* genome and is controlled by a *flaA* promoter (P_FlaA_). TetR binds to *tetO1* blocking expression of gGT. The inducer anhydrotetracycline (ATc) binds to *tetR* resulting in disassociation of the repressor and activation of *ggt* expression. (b) gGT-activity of conditional gGT mutants. Bacteria were cultured in BHI/10% FCS in the presence of 100 ng/ml ATc for 24 h. *H. pylori g:O1 t* has *tetO1* and *tetR* integrated into its genome. In *H. pylori g:O1* C only the *ggt* promoter was replaced by *tetO1*, *n* = 3–6. Bars represent the mean of three to six independent experiments as represented by single dots. Error bars represent the standard deviation. n: number of independent experiments. Mann-Whitney U-test. *p < 0.05.

*H. pylori* strains with conditional gGT activity were generated using the tetracycline-inducible gene expression system developed by Debowski et al.^33–35^ (Figure 1(a,b), Figure S1 a). The correct insertion of the tet repressor (*tetR*) into the genome and the exchange of the *ggt* promoter by the tet inducible promoter tetO1 was verified by PCR (Figure S1(b)) The system’s functionality was assessed by measuring gGT activity and protein expression in the presence and absence of the inducer anhydrotetracycline (ATc). Expression of gGT was determined to be 1.5- to 2-fold higher in this new model than in the corresponding wt strains (Figure 1(b)). In addition to the inducible strain (*g:O1 t*), a control strain (*g:O1 C*) lacking the tetracycline repressor (*tetR*) was developed, resulting in constitutively high gGT activity to serve as a control in experiments with the inducible system. In the absence of the inducer, no protein was detected in Western blots (Figure S1 c), and gGT enzymatic activity remained below detection limits (Figure 1(b)), confirming that *tetR* tightly represses *ggt* expression.

Key parameters for the inducible system, including inducer concentration and induction/silencing times, were determined. Low ATc concentrations (5 ng/ml for the G27 strain and 25 ng/ml for the PMSS1 strain) were enough to fully induce gGT expression (Figure S1(d)). However, the developers of the system recommended an ATc concentration of 100 ng/ml to fully induce strains.^33^ Thus, after confirming that this inducer concentration does not significantly influence bacterial growth compared to the solvent control (Figure S1(e)), this concentration was selected for further experiments. Using this inducer concentration, maximum induction of gGT activity can be achieved in the PMSS1 strain within 18 h and in the G27 strain within 6 h. Similarly, silencing of *ggt* expression can be reached after 8 h or 6 h, respectively (Figure S1(f)).

In summary, these results demonstrate the successful generation and characterization of functional *H. pylori* tet-on gGT strains, providing a valuable model for controlled studies of gGT activity.

### *H. pylori* gGT is important for initial colonization and for persistence

**Figure 2.**
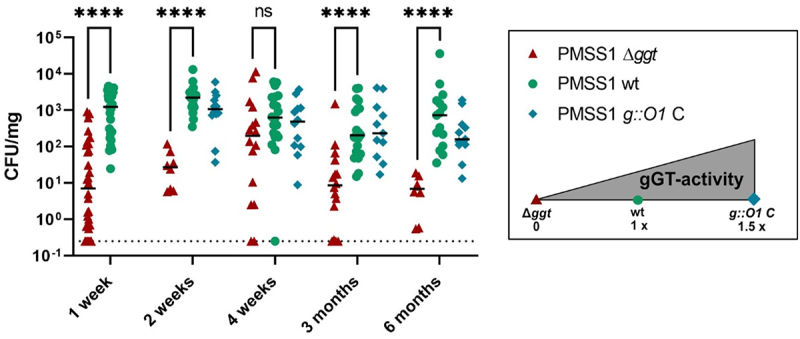
*H. pylori* gGT contributes to initial colonization and persistence colony forming units (CFU) in the stomach (mg) of C57BL/6 mice after infection with PMSS1 wt, ∆*ggt* or *g:O1 C*. Mice were inoculated with 2 × 10^8^ bacteria twice and were sacrificed after a defined amount of time (1 week −6 months) after the first infection. Colonization was assessed by plating stomach homogenates. The detection limit is indicated by a dotted line. Each dot represents one individual mouse. PMSS1 wt (*n* = 15–27), PMSS1 ∆*ggt* (*n* = 8–32), PMSS1 *g:O1* C (*n* = 12), n: number of mice. Mann-Whitney U-test. ****p < 0.01.

*H. pylori* gGT is a virulence factor that facilitates initial colonization.^8,11,12^ However, Wüstner et al. observed similar infection levels of a wt and a gGT-deficient strain in a mouse model after 1 month of infection, despite an initial colonization hurdle of the gGT-deficient strain.^8^ This raised the question during which infection phases gGT is important for *H. pylori* colonization. To address this question, mice were infected for up to 6 months either with a wt-, a gGT-deletion strain or a strain expressing high levels of gGT (*g:O1 C*) (Figure 2). At the beginning of the infection, colonization was significantly lower in mice infected with the gGT deletion strain compared to those infected with PMSS1 wt and PMSS1 *g:O1 C*, confirming previous findings about the role of gGT during initial infection. However, colonization levels of the gGT deletion strain increased over time, peaking after one month of infection, consistent with observations by Wüstner et al. Subsequently, after this colonization peak, infection levels in mice infected with PMSS1 ∆*ggt* decreased, indicating that gGT also contributes to persistence. After 3 months and 6 months of infection colonization levels of the gGT-deficient strain were significantly lower than those of the wt and gGT-high strain. No difference in colonization was observed comparing the infection levels of mice inoculated with the wt or high gGT-expressing strain throughout the course of the infection.

*H. pylori* gGT has been shown to impact the infiltration of neutrophils and CD8^+^ T cells,^8^ and to modulate CD4^+^ T cell proliferation and function.^9^ Thus, the infiltration of these immune cells was detected in infected tissues to explore whether gGT-deficient bacteria differ in their ability to recruit immune cells and cause pathology. (Figure S2). After 4 weeks post-infection the number of CD8^+^ T cells was lower in the stomach of mice infected with the gGT deficient strain, as previously observed.^8^ In contrast, differences in CD4^+^ T cell recruitment were observed 6 months post-infection.

In summary, these findings suggest that gGT is not only crucial for initial colonization but also for persistence.

### *H. pylori* gGT contributes to acid resistance supporting *in vivo* colonization

**Figure 3.**
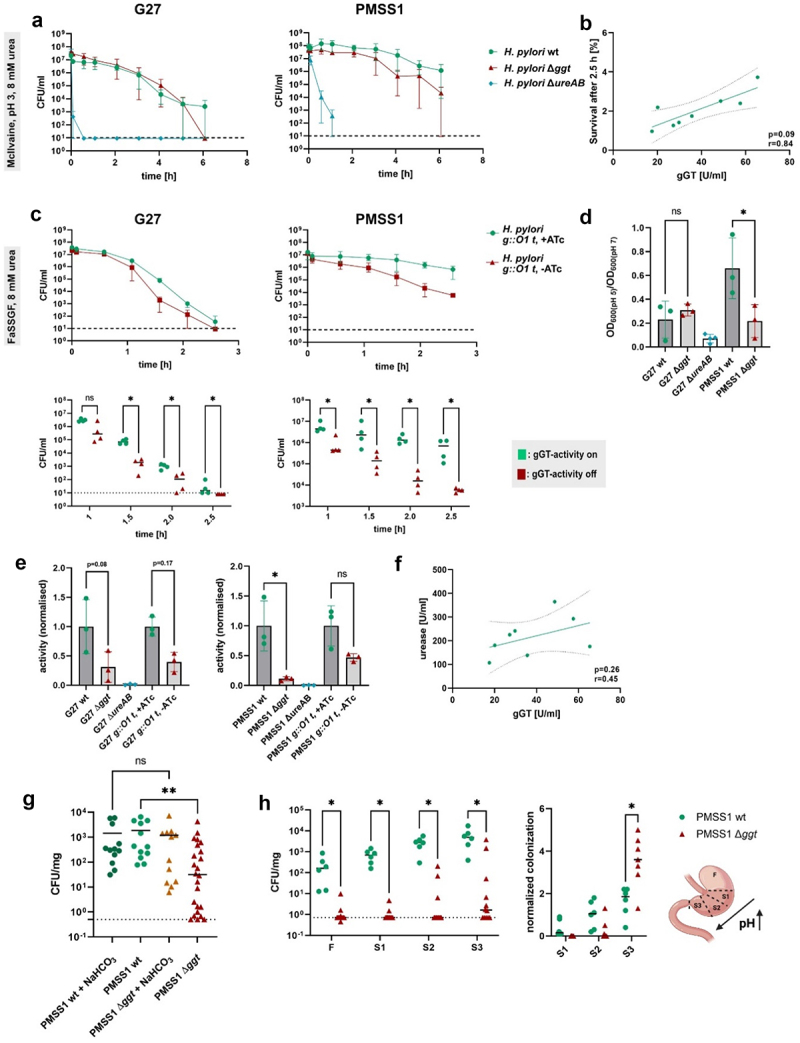
*H. pylori* gGT contributes to acid survival supporting stomach colonization (a) Survival of *H. pylori* in McIlvaine buffer (pH 3) supplemented with 8 mm urea, *n* = 3. The detection limit of the assay is indicated by a dotted line. (b) Correlation of survival of clinical isolates after 2.5 h with gGT-activity. Survival was determined in McIlvaine buffer (pH 3) supplemented with 8 mm urea. Each dot represents the mean survival of one clinical isolate, *n* = 3. (c) Survival of *H. pylori* in fasted state simulated gastric fluid (FaSSGF) supplemented with 8 mm urea. The detection limit of the assay is indicated by a dotted line., *n* = 4. (d) Growth of *H. pylori* in Brucella broth supplemented with 20% FCS at pH 5 normalized to the growth in Brucella broth at pH 7. Growth was quantified spectrophotometrically after 30 h. *n* = 3. (e) Urease activity after exposure of *H. pylori* to pH 5. Strains were grown in BHI with 10% FCS, resuspended for 2 h in McIlvaine buffer (pH 5) and lysed for the detection of urease activity. Data was normalized to the mean activity of H. pylori wt or tet-on H. pylori g:O1 t. *n* = 3. (f) Correlation of urease- and gGT-activity of clinical isolates. Each dot represents the mean activity of an isolate. *n* = 3. (g, h) Colony forming units (CFU) in the stomach (mg) of C57BL/6 mice after infection with PMSS1 wt or ∆*ggt*. Mice were inoculated with 2 × 10^8^ bacteria. Colonization was assessed by plating stomach homogenates. The detection limit is indicated by a dotted line. Each dot represents one individual mouse. (g) CFU/mg after 1 week of infection. Two groups of mice received sodium bicarbonate (NaHCO_3_) before the infection to elevate the pH in the stomach. (h) CFU/mg in different stomach sections (F: forestomach, S1–S3: stomach sections) after infection for 2 weeks. Colonization was normalized to the mean CFU/mg across sections S1–S3 for each mouse. n: number of independent experiments. Pearson’s correlation (b, f); Mann-Whitney U-test (c, g, h); ANOVA (d, e) *p < 0.05, **p < 0.01.

Next, we hypothesized, based on the study by Miller and Maier, that gGT is important for acid resistance which might contribute to the effect of this virulence factor on initial colonization.^14^ *H. pylori* gGT was described to enhance the survival of strain 26695 at pH 3 in the presence of high urea concentrations.^14^ However, this study does not fully account for the complexity of the human stomach, where the gastric fluid pH ranges from 1.7 to 6 depending on food intake,^36^ and additional components of the gastric fluid such as the proteolytic enzyme pepsin^37^ or the bile acid sodium taurocholate^38^ further challenge *H. pylori* acid survival and growth. Consequently, simulation of stomach-like conditions is important to understand the role of gGT in acid survival. First, survival assays were performed with a physiologically relevant urea concentration of 8 mm.^39,40^ Under these conditions, both wt G27 and PMSS1 exhibited enhanced survival rates compared to isogenic gGT-deficient strains (Figure 3(a)), confirming previous observations by Miller and Maier using a urea concentration of 20 mm.^14^ PMSS1 wt demonstrated robust survival for up to 6 h, while the viability of the gGT-deficient mutant began to diminish after 2 h. The G27 strain was more vulnerable to acidity compared to PMSS1 under these conditions. Here, viable wt bacteria persisted even after 6 h, but no gGT-deficient mutants could be recovered after the same time interval (Figure 3(a)). To confirm that McIlvaine buffer at pH 3 with 8 mm urea poses an acidic challenge to *H. pylori*, urease-deficient strains, which are well-known to be highly sensitive to acidic conditions,^41^ were used as internal controls that died within an hour in this milieu as expected. Moreover, to rule out that gGT’s impact on acid resistance was due to a general survival defect of gGT-negative strains in McIlvaine buffer, survival was also monitored at pH 5 (Figure S3 a). At this pH, the urease-deficient control strains and the gGT-deficient knock-out strains survived equally well as the wt for at least 4 h. To investigate whether the effect of gGT on acid resistance depends on the presence of urea, survival at pH 3 was assessed without supplements (Figure S3 b). A supporting effect of gGT on *H. pylori’s* acid survival was evident but was less pronounced than in the presence of urea. After assessing the effect of gGT on acid survival with the strains PMSS1 and G27, the survival of fresh clinical isolates with different gGT-activities was measured, revealing a significant correlation between gGT-activity and acid survival at pH 3 in McIlvaine buffer supplemented with physiological concentrations of urea (Figure 3(b)).

Next, we monitored survival under conditions similar to the gastric environment. For this purpose, fasted-state simulated gastric fluid (FaSSGF) that includes constituents like pepsin, low concentrations of bile salts, and lecithin in physiological concentrations was used. FaSSGF has a comparable pH, osmolarity, and surface tension^27,42^ as the gastric fluid in the fasted state. G27 showed reduced survival rates in this medium compared to PMSS1, however, differences in survival between wt and gGT-deficient strains starting after 1.5 h showed that gGT-activity significantly supported survival of *H. pylori* strains PMSS1 and G27 in FaSSGF. (Figure 3(c) and Figure S3(c)).

To infect the host *H. pylori* needs to survive the harsh environment of the stomach lumen for a short period. However, after arriving at the gastric mucosa, the bacterium is replicating in a rather neutral environment. Nevertheless, there is a pH gradient along the mucosa^39^ and the mucosal pH can drop after food intake.^43^ Thus, *H. pylori* has developed mechanisms enabling growth at mildly acidic conditions.^44,45^ To analyze the function of gGT in this context, we monitored *in vitro* growth of *H. pylori* strains G27 and PMSS1 in the presence and absence of gGT at pH 5. BHI medium with 20% FCS did not allow for growth of *H. pylori* in these conditions (Figure S4 a), but Brucella medium supplemented with 20% FCS supported growth of PMSS1 and G27. A low pH reduced growth of the wt strains (to 80% for PMSS1, to 20% for G27) compared to neutral conditions. *H. pylori* gGT strongly supported growth of PMSS1 while there was no observable effect of gGT on the strain G27 in the tested conditions. (Figure 3(d)). In addition, growth of wt strains was accompanied by a strong increase in the pH of the medium while strains deficient of gGT elevated the medium pH to a lower extent (Figure S4 b).

Urease activity is a central mechanism of *H. pylori* enabling the survival^46^ and growth^44^ of the bacterium at low pH by catalyzing the hydrolysis of urea to ammonia and carbon dioxide. As gGT supports acid survival (Figure 3(a–c)) and contributes to pH increase in the presence of urea (Figure S3 d), the reduced survival in the absence of gGT-activity could be induced by impaired urease activity. To test this hypothesis, we evaluated urease activity after exposing *H. pylori* to pH 5 in McIlvaine buffer. Urease is considered to be a cytoplasmic protein, but 10–30% of the enzyme is also reported to be localized on the bacterial surface.^47,48^ Urease activity was notably reduced by 50% in *H. pylori* lysates (Figure 3(e)) and in the supernatants (Figure S3 E) in gGT deletion strains compared to wt strains.

Since both wt and gGT-deficient strains exhibited comparable survival at pH 5 in McIlvaine buffer (Figure S3 a), variations in urease activity cannot be attributed to different survival modalities of wt and gGT-deficient strains. The reduction in urease activity was found in deletion strains and in the *H. pylori g:O1 t* model in the absence of ATc, suggesting a functional link between urease and gGT. This diminished urease activity was accompanied by a lower amount of this enzyme present in the supernatants of gGT-deficient *H. pylori* strains and, in the case of the strain G27, by a lower urease concentration in the lysate, as detected by Western blot (Figure S3 f) and proteome analysis (Figure S5). However, in the PMSS1 lysate, reduced urease activity could not be linked to decreased protein expression, suggesting other control mechanisms. To assess whether the activity level of gGT, in addition to its presence, is important for urease function, urease activity was assessed in clinical isolates. Urease activity tended to correlate with gGT-activity, even though this correlation was not found to be significant (Figure 3(f)).

Taken together, our data revealed a protective function of gGT in acidic environments, supporting bacterial survival and growth. To link these findings to *in vivo* colonization, we used a murine infection model in which C57BL/6 mice were administered sodium bicarbonate by oral gavage prior to infection to elevate the gastric pH. Following the infection of mice with wt or gGT-deficient PMSS1, colonization levels were assessed by plating stomach homogenates. While the wt strain did not benefit from sodium bicarbonate treatment, the infection level of the gGT-deficient strain notably increased after the treatment, overcoming the initial colonization hurdle of gGT-deficient PMSS1 (Figure 3(g)). Since the pH in the murine stomach varies by anatomical location – low in the corpus and more alkaline toward the duodenum^49^ – the acid-sensitive gGT-deficient strain might show a colonization pattern different from that of the wt strain. To examine this, colonization levels were measured in different stomach regions (Figure 3(h)). Both PMSS1 wt and PMSS1 ∆*ggt* preferentially colonized the antral part of the stomach. However, the gGT-deficient strain showed a more restricted colonization of the antrum, while the wt strain could colonize the corpus region at high levels as well.

Thus, our findings collectively indicate that gGT supports *H. pylori* colonization in acidic environments, partly by maintaining urease activity.

### Absence of *H. pylori* gGT leads to an upregulation of proteins involved in nitrogen and iron metabolism

Even after elevation of the pH in the stomach, the colonization levels observed in mice infected with the gGT-deficient strain still tended to be lower than in mice infected with the wt strain (Figure 3(g)), indicating that gGT might also support colonization by other mechanisms independent from acid resistance. To identify plausible additional functions of gGT or compensatory mechanisms allowing gGT-deficient bacteria to colonize, isolates retrieved from the murine stomach were compared to the aliquots initially used for infection. The infection levels of mice used for this experiment followed the same trend as described in the previous section (Figure S6 a).

To study protein expression profiles, re-isolates retrieved from different mice after 1 week, 1 month or 3 months of infection and the strains initially used for infection were grown until the early stationary phase (Figure S6 b), lysed and analyzed by liquid chromatography-mass spectrometry (LC-MS/MS).

Alterations in the expression levels of various proteins implicated in the nitrogen metabolism were observed when comparing the proteome of PMSS1 wt and PMSS1 ∆*ggt*. *H. pylori* employs several enzymes to produce ammonia, which is subsequently assimilated by the bacterium.^50^ This enzymatic ensemble encompasses gGT, formamidase (AmiF), amidase (AmiE), urease (UreAB), aspartate-ammonia lyase (AspA) and asparaginase (AsnA). Notably, gGT-deficient strains showed significantly elevated expression levels of both AmiF and SdaA and a discernible but not significant increase in the expression of AmiE (Figure S7 a). The altered expression of AmiE and SdaA was present pre-infection and remained to be similarly different between wt and gGT-deficient strains after infection. However, AmiF expression diminished during the infection phase.

To confirm the changes in expression levels of ammonium producing enzymes detected by mass spectrometry, AmiE and AmiF activities were measured in the presence and absence of gGT in the strain PMSS1. Both enzyme activities were increased 1.5- to 2-fold in the absence of gGT, when gGT was either deleted or its expression repressed by TetR (Figure S7 b). Moreover, enzymatic activity assays strengthened the finding that AmiF activity is reduced in re-isolates, while AmiE activity remained unchanged (Figure S7 c). To understand whether altered AmiF and AmiE activities in the absence of gGT generally apply to *H. pylori* or whether this effect is strain-specific, activity measurements were also conducted with the G27 strain. Analogous to the observations in PMSS1, AmiF activity tended to be increased in the G27 strain in the absence of gGT. However, the effect of AmiE differed in G27, as the enzymatic activity was decreased upon silencing or deletion of gGT (Figure S7 b).

In concert with ammonium-producing enzymes, peptide transporters ensure the supply of the bacterium with nitrogen sources.^50^ In this context, we detected differential expression of OppA, an oligopeptide transporter with unknown specificity in *H. pylori*^51^ (Figure S7 a). OppA expression was found to be significantly higher in gGT-deficient compared to wt re-isolates subsequent to infection. In conclusion, the absence of gGT prompts *H. pylori* to orchestrate adjustments in expression profiles of various proteins implicated in nitrogen assimilation, indicating a central function of gGT in the nitrogen metabolism.

**Figure 4.**
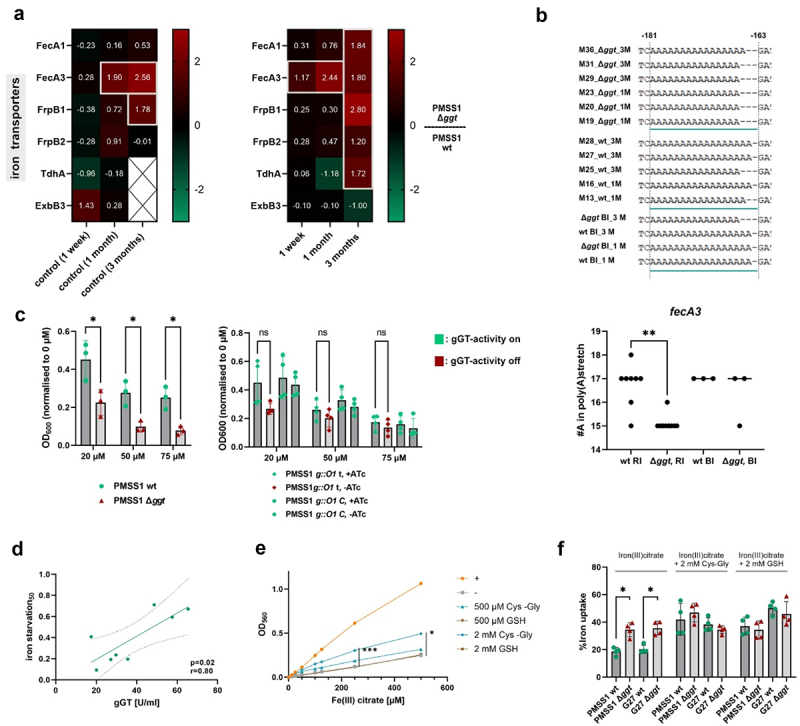
*H. pylori* gGT promotes survival in iron-restricted environments (a) Log_2_ transformed fold change of proteins potentially involved in metal acquisition comparing the proteome of PMSS1 wt and ∆*ggt*. The proteomes of three single clones isolated after 1 week, 1 month, and 3 months of infection from different mice were determined. Significant differences (-log_10_(p-value) > 1.3 and log_2_(fold change) ≥ I1I) are marked with gray boxes. Missing data points are colored white and are marked with a cross. (b) Schematic representation of the poly(a)tract in the *fecA3* upstream region between positions − 181 and − 163 before the translation start. The number of adenine bases in the tract was quantified for all re-isolates (RI) and for the aliquot used for infection (BI) (c) Growth of *H. pylori* in iron-restricted medium. Deferoxamine mesylate (DFO) was added to the culture in different concentrations (20 µm, 50 µm, and 75 µm). Growth was monitored spectrophotometrically and was normalized to the growth without supplementation. Bars represent the mean of three to four independent experiments as indicated by dots. Error bars represent the standard deviation. (d) Correlation of growth in iron-limited conditions with gGT-activity. Each dot represents the mean growth of one clinical isolate as measured in three independent experiments. Clinical isolates were grown in the presence of 50 µm DFO and growth data were normalized to the growth without supplementation. (e) Reduction of iron(iii)citrate to Fe^2+^ in the presence of varying concentrations of Cys-Gly or GSH. As a positive control (+) 1 M ascorbic acid was added to fully reduce the available iron. As a negative control (-) Fe^2+^ concentrations were measured in a Fe(III) citrate solution. (f) Uptake of iron(iii)citrate into *H. pylori* in the presence and absence of 2 mm GSH or Cys-Gly. Students t-test (a, c, e, f). ANOVA (c); Pearson’s correlation (d). *p < 0.05.

In addition to the alterations in the expression of proteins associated with the nitrogen metabolism, our experiment also revealed differential expression of iron-assimilating proteins in the absence of gGT. Among these is FecA3. The absence of gGT led to an upregulation of FecA expression, which is a potential iron-citrate transporter,^52^ both pre- and post-infection (Figure 4(a)). Furthermore, significantly increased expression of other proteins associated with iron acquisition, including the potential iron(III)citrate transporter FecA1, heme transporters FrpB1 and FrpB2 and the TonB-dependent heme receptor A (TdhA), was evident in gGT-deficient mutants after 3 months of infection (Figure 4(a)). Genome sequencing did not reveal an immediate explanation for most of these findings. However, the length of a poly(A) tract in the *fecA3* upstream region was found to be significantly longer in wt compared to gGT-deficient isolates (Figure 4(b)) . This length variable tract might be involved in regulation of the gene as other pathogenic factors of *H. pylori* like the adhesin *sabA*^53^ or the small RNA *nikS*^54^ are already known to be regulated by similar patterns. However, in these examples the variable stretch is found closer to the transcription start.

These findings, together with the prediction that gGT is controlled by the central iron regulator Fur,^55^ raised the question whether gGT plays a role in the bacterial iron metabolism. To address this question, we cultivated *H. pylori* strains under iron-restricted growth conditions with varying concentrations of the iron chelating agent deferoxamine mesylate supplemented to the growth medium. This experiment revealed that gGT-proficient strains grew significantly better under iron limiting conditions compared to their isogenic gGT-deficient mutant strains (Figure 4(c)). Moreover, the gGT-activity of clinical *H. pylori* isolates correlated with the ability to grow in iron-limited conditions (Figure 4(d)). The observed upregulation of iron acquisition proteins in gGT-deficient mutants might seem counterintuitive in this context. However, Pich et al. hypothesized that gGT activity is involved in reducing Fe^3+^ to Fe^2+52^, which is subsequently assimilated by the FeoB transporter.^56^ To further investigate this hypothesis, Fe^2+^ release from Fe(III)citrate in the presence of Cys-Gly was measured. Indeed, at concentrations of 500 µM and 2 mm, Cys-Gly released significantly more Fe^2+^ compared to equivalent concentrations of glutathione (Figure 4(e)). This suggests that Cys-Gly might make Fe^2+^ available to the bacterium. To assess how this affects iron uptake of *H. pylori*, iron absorption of wt and gGT-deficient strains in the presence of Cys-Gly or glutathione was measured. Both G27 ∆*ggt* and PMSS1 ∆*ggt* strains showed a 1.8-fold enhanced iron (III)citrate uptake compared to their respective wt strains (Figure 4(f)). However, this difference was no longer observed when Cys-Gly or GSH was added, indicating that these compounds can support the wt bacteria to take up certain iron sources. Taken together with the protein expression data (Figure 4(a)), the results suggest that the elevated expression of specific proteins related to iron-acquisition could act as a compensatory mechanism in gGT-deficient strains, facilitating increased Fe^3+^ uptake while wt strains might rely more on Fe^2+^.

In summary, our findings suggest that gGT confers a growth advantage in iron-deprived environments, potentially by releasing Fe^2+^. This effect might be partially counterbalanced in gGT-deficient strains by an increased uptake of Fe^3+^ under iron rich conditions.

### Flagellar motility and adhesion capacity are enhanced in the absence of gGT subsequent to infection

In addition to its impact on the expression of proteins related to the bacterial metabolism, the absence of gGT also affected bacterial traits that have not been linked to gGT activity yet, including flagellar proteins and adhesion factors.

**Figure 5.**
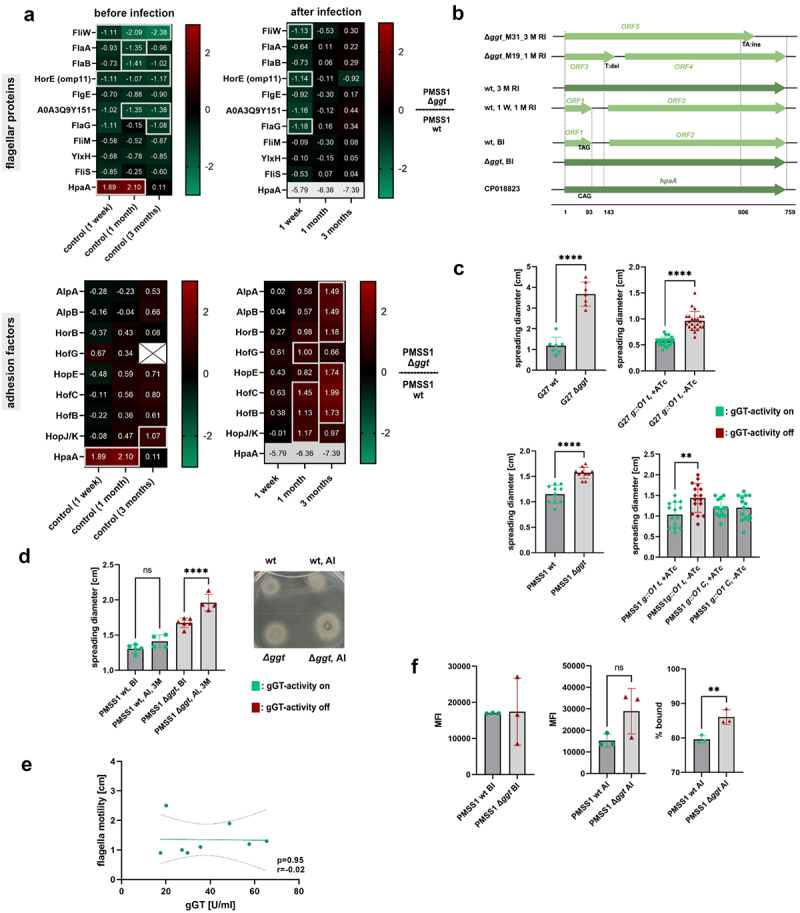
*H. pylori*’s motility and adhesion capacity is increased in the absence of gGT during infection (a) Log_2_ transformed fold change of flagellar proteins and adhesion factors comparing the proteome of PMSS1 wt and ∆*ggt*. The proteomes of three single clones isolated after 1 week, 1 month, and 3 months of infection from different mice were determined. Significant differences (-log_10_(p-value) > 1.3 and log_2_(fold change) ≥ I1I) are marked with gray boxes. Missing data points are colored white and are marked with a cross. Values out of the range of the heat map are colored gray. (b) Schematic representation of the genomic organization of the *hpaA* locus as determined by whole genome sequencing. ins: insertion, del: deletion. ORF: open reading frame, BI: before infection, RI: re-isolate (c,d) Flagellar motility of *H. pylori* strains in the presence and absence of gGT. Soft agar assays on Brucella/10% FCS plates were performed. 100 ng/ml ATc was added to the plates to switch on gGT-activity in inducible strains. gGT-activity was not induced in the pre-culture prepared for the experiment. Motility of deletion strains and inducible strains was determined (c) as well as motility before (BI) and after infection (AI) (D). Bars represent the mean of 8 to 14 independent experiments as indicated by single dots (C) or the mean motility of 4 to 5 isolates as measured in three independent experiments (d). (e) Correlation of flagellar motility of clinical isolates with gGT-activity. Each dot represents the mean motility of an isolate as determined by 3 independent experiments (f) Adhesion capacity of *H. pylori* before infection (BI) and after infection (AI) to AGS cells. Bars represent the mean of three independent experiments. MFI is the median fluorescence intensity on the PE channel. Error bars represent the standard deviation. Student’s t-test (a, c, f). ANOVA (d), Pearson’s correlation (e). **p < 0.01, ****p < 0.0001.

Comparative analysis of the proteome of wt and gGT-deficient PMSS1 prior to infection revealed an increase of flagellar proteins in the wt strain (Figure 5(a)). Mainly, proteins of the flagellar filament, including flagellin A (FlaA), B (FlaB), and flagellar hook proteins, including FlgE, were found to be affected by the absence of gGT. Increased secretion of the same flagellar proteins was observed for *H. pylori* G27 and PMSS1 ∆*ggt* mutants, as compared to the respective wt strains after exposure to pH 5 (Figure S5), implying that the observed lower presence of flagellar proteins in the cytoplasm of the mutant strains may result from a difference in secretion.

After infection, substantial variations in flagellar proteins between wt and gGT-deficient strains were observed only in isolates obtained after one week of infection, but not in re-isolates from later infection time points. This suggests that expression or secretion of flagellar proteins shifts during the course of infection in gGT-deficient mutants that are able to colonize mice. These flagellar proteins included the flagellar assembly factor FliW, flagellins FlaA/B, flagellar hook protein (FlgE), flagellar accessory protein FlaG, and OMP11, which was hypothesized to be specific for the flagellar sheath membrane.^57^ Notably, only the expression of the flagellar sheath associated protein HpaA displayed a distinct pattern, while expression of other flagellar proteins followed the same trend during infection. Increased levels of HpaA expression were detected prior to infection in the gGT-deficient mutant, which was found to be significantly decreased (60-fold) in gGT-deficient re-isolates. We confirmed these results by Western blot (Figure S8). Here, PMSS1 wt exhibited stronger expression of HpaA before infection, while its expression was reduced in re-isolates. In contrast, the wt strain showed lower expression of HpaA compared to isogenic gGT-deficient strains after infection. In addition, genome sequencing revealed that the observed differences can be traced to the genomic level (Figure 5(b)). The strains used for infection already exhibited divergence or heterogeneity in the *hpaA* gene. In the wt strain before infection, *hpaA* was disrupted by a mutation that created a stop codon while the gGT-deficient strain had a fully functional gene. However, during infection, some wt bacteria regained the undisrupted open reading frame, whereas all gGT-deficient re-isolates showed a disrupted *hpaA* gene. This suggests a strong selection for the inactivation of this gene *in vivo* in the absence of gGT. This disruption of *hpaA* in PMSS1 ∆*ggt* re-isolates resulted from different insertions or deletions within the gene. Figure 5(b) depicts the gene organization of two exemplary gGT-deficient re-isolates.

To investigate whether the observed differences in flagellar proteins affected motility, soft agar assays were performed. Strikingly, deletion or silencing of gGT in both G27 and PMSS1 strains significantly increased motility (Figure 5(c)). Specifically, motility of the gGT-deficient G27 strain was 3-fold higher compared to the isogenic gGT-proficient counterpart, while motility of PMSS1 increased 1.5-fold in the absence of gGT. In a next step, we assessed the effect of infection-induced changes in flagellar protein expression on motility. Therefore, motility of re-isolates was compared to the strains initially used for infection. The motility of PMSS1 wt remained unaltered after infection, whereas the gGT-deficient re-isolates gained motility in comparison to the strain initially used for infection (Figure 5(d)). To determine whether gGT-activity correlates with motility, flagellar movement of clinical isolates with different gGT-activities was determined. Our findings suggest that there is no correlation between gGT-activity and flagellar motility and that other factors might play a more dominant role in controlling bacterial movement (Figure 5(e)).

Collectively, these findings suggest that absence of gGT prompts *H. pylori* to differentially regulate flagellar proteins, resulting in an increased motility of gGT-deficient strains, a trait that becomes even more pronounced during infection.

Furthermore, the absence of gGT during infection caused the emergence of re-isolates characterized by an increased expression of adhesion factors. While AlpA, AlpB^58^ and HorB^59^ are known to support *H. pylori* binding to gastric epithelial cells, other outer membrane proteins from the Hof- and Hop family including HofG, HopE, HofC, HofB and HopJ/K, affected by the absence of gGT, are only predicted to contribute to binding.^60^ In comparison to the wt strain, only putative adhesins HpaA and HopJ/K displayed increased expression in the gGT-deficient mutant prior to infection, while re-isolates retrieved after 1 month or 3 months of infection exhibited substantially elevated expression levels of several known and putative adhesion factors (Figure 5(a)). To dissect whether the variations detected by mass spectrometry result in alterations of the binding capacity of *H. pylori*, TAMRA-stained bacteria were added to AGS cells and the bacterial signal on AGS cells was quantified using flow cytometry. While strains initially used for infection displayed comparable binding profiles independently of gGT, gGT-deficient re-isolates exhibited an increased binding capacity to the gastric epithelial cell line as compared to wt re-isolates. In summary, these findings indicate that the absence of gGT during infection favors the selection of clones with higher adhesion capabilities (Figure 5(f)).

Taken together, comparative analysis of the proteome of wt and gGT-deficient PMSS1 revealed that gGT-deficiency has a considerable effect on *H. pylori* to differentially express various bacterial proteins, encompassing metabolic enzymes, flagellar proteins, and adhesion factors.

## Discussion

*H. pylori* possesses a repertoire of virulence factors that enables its survival in the hostile stomach environment, allowing for persistent colonization of the gastric mucosa. In this study, we investigated the highly conserved virulence factor gGT and linked its activity to other bacterial traits important for successful colonization including acid survival, iron acquisition, motility, and adhesion.

Several studies have indicated the contributory role of *H. pylori* gGT to initial colonization.^8,11,12^ However, the specific functional roles of the enzyme underlying its effect on initial colonization have remained elusive. Since an effect of gGT on the immune system was largely ruled out in this context,^8^ as colonization differences emerge prior to the infiltration of innate or adaptive immune cells, a metabolic advantage provided by the enzyme became a plausible explanation for the reduced colonization ability of strains deficient for gGT.

We found evidence that gGT contributes to bacterial survival in physiologic fasted state-like low pH conditions by supporting the function of the key enzyme urease. Although Miller and Maier^14^ proposed a role of gGT for acid resistance, our study extends their findings by simulating different physiological acidic environments and, more importantly, by providing a new mechanism for the contribution of gGT to acid resistance, by linking it to urease activity. However, the functional relationship between gGT and urease activity is still unknown. Miller and Maier hypothesized that urea-derived ammonia was assimilated in the bacterial cytoplasm into nitrogen-containing molecules that are deaminated in the periplasm. In the absence of gGT, deamination of glutamine in the periplasm is abolished, potentially affecting ammonia assimilation in the cytoplasm, which would result in high concentrations of toxic ammonia and might ultimately cause *H. pylori* to limit its urease activity. Moreover, nickel is an essential cofactor for urease function.^61^ Considering that host factors like lactoferrin can bind nickel in addition to iron^62^ and that *fecA3* might be involved in the uptake of complexed nickel ions,^63^ an effect of gGT on nickel uptake also seems plausible which might affect urease function in gGT-deficient strains.

In addition to refining the role of gGT in acid resistance *in vitro*, we were able to directly link gGT-mediated acid resistance to initial colonization *in vivo*, confirming a pivotal role of gGT in the acidic stomach environment. In humans, *H. pylori* seems to depend even more on gGT for infection than in the mouse model. While all human isolates express this enzyme,^13^ mouse infections with gGT-deficient strains are possible.^8,12^ This intriguing difference may be attributed to the markedly lower pH levels in the human stomach, which are reported to be pH 1.7 in the fasted state,^64^ whereas the fasted murine stomach maintains a relatively less acidic pH of 4.^65^

Apart from being important to cope with acidity and thereby supporting initial colonization, it is very likely that additional functions and molecular mechanisms related to gGT activity play a role in colonization and persistence because, even after elevation of the stomach pH, the gGT-deficient bacteria were still not able to colonize at the same level than the wt, and over time, reduced bacterial load was observed in mice infected with the gGT knockout strain. Those alternative mechanisms include alterations in important metabolic pathways, changes in flagellar proteins as well as differential regulation of molecules important for adherence to the gastric epithelium.

During infection, the host actively restricts iron availability for pathogens as part of the innate defense to limit growth of invading microbes.^66^ *H. pylori* genes essential for iron acquisition, like *feoB*^56^ or *fecA1*,^67^ for iron storage like *pfr*^68^ or for the regulation of the iron metabolism, like *fur*,^69^ were reported to strongly support *H. pylori* colonization. Notably, we observed that gGT-deficient mutants are highly sensitive to iron-restricted environments, making it very likely that a hampered iron acquisition in the absence of gGT would be another mechanism contributing to the reduced colonization capacity of the gGT-deficient strain. Although a direct functional link of gGT to iron assimilation has not been described for *H. pylori* yet, *H. pylori* gGT is predicted to be Fur regulated and thus involved in the iron metabolism of the bacterium.^55^ Pich et al. already proposed a plausible hypothesis for the function of *H. pylori* gGT in iron acquisition based on findings in the mycopathogen *Histoplasma capsulatum*.^52^ According to this hypothesis, Cys-Gly, produced by gGT-activity, reduces Fe^3+^, which is typically bound in complex molecules, resulting in soluble Fe^2+^ ions that can be taken up by *H. pylori*. This mechanism was already shown to enable *H. capsulatum* to use hemin and transferrin as iron sources because gGT-dependent production of Cys-Gly causes the release of iron from these sources.^70,71^ Our observation that iron uptake in the wt strain is enhanced in the presence of Cys-Gly, suggests that this mechanism might also be important for *H. pylori’s* iron acquisition. Nevertheless, further studies are needed to link the contribution of this mechanism to *H. pylori* colonization.

Apart from iron, the nitrogen metabolism is also crucial to support *H. pylori* growth in the stomach. *In vitro* studies reported gGT to be important for the bacterial nitrogen metabolism by hydrolyzing glutamine which is followed by the uptake of glutamate via the transporter GltS.^13^ The discovery of Kavermann et al. demonstrating the crucial role of this transporter for *H. pylori* colonization of Mongolian gerbils,^72^ strongly suggests that this gGT-dependent pathway is also important *in vivo*. In addition, the dependency of *H. pylori* on this metabolic pathway *in vivo* is supported by the observed upregulation of ammonium-producing enzymes and peptide transporters that might help the bacterium to acquire nitrogen-containing molecules in the absence of gGT by other means than glutamine hydrolysis.

Additionally, nutrient limitation can trigger flagellar synthesis,^73^ while a low cellular energy status can activate chemotaxis via the sensor TlpD.^74^ Consequently, potential nutrient or energy shortages in the absence of gGT might increase bacterial motility, as suggested by our proteome analyses, enabling *H. pylori* to actively search for alternative nutrient sources and supporting thereby bacterial colonization. The fact that several proteins involved in flagellar *H. pylori* motility including the flagellar proteins flagellin A (FlaA), flagellin B (FlaB)^75^ and FlgK^76^ and the flagellar motor protein (MotB)^77^ were already shown to be essential to attain robust colonization, further stresses the importance of this bacterial trait during infection.

In parallel, the increased levels of adhesion factors observed in the absence of gGT would favor a tighter bacterial adhesion to host cells. Although an upregulation of flagellar proteins and adhesins at the same time seems counterintuitive, one may hypothesize that the absence of gGT might force the bacterium to move to other infection sites to acquire nutrients, and as result *H. pylori* needs to interact with the epithelium more frequently. The presence of the adhesion factors AlpA, AlpB and HorB are well-known to be beneficial for *H. pylori* colonization,^59,78^ suggesting that an increase in their expression during infection, as observed here, can be supportive for bacterial colonization. In addition, gGT-dependent alterations in the protein HpaA, as detected in this study, can also impact colonization levels because HpaA was found to be essential for *H. pylori* infection of mice^79^ and Mongolian gerbils.^72^

In addition to its supportive role during initial colonization, we observed that *H. pylori* gGT also supported bacterial persistence in the stomach after chronic infection (3 and 6 months). In line, Oertli et al.,^7^ showed almost complete clearance of gGT-deficient strains from the murine stomach after two months of neonatal infection. In contrast, this clear effect was not previously detected after infection of adult mice for 6 months, which could be explained by the use of higher infectious dose and mice from a different origin.^8^ Considering the rapid adaptations of *H. pylori* to the environment, these slight differences could have an impact on the levels of colonization.

We have uncovered several novel mechanistic functions of *H. pylori* gGT through the analysis of re-isolates from a mouse infection model. Although findings were validated with clinical isolates, indicating their relevance in the human context, it is important to note that using a mouse model may potentially limit the functional mechanisms of gGT that can be detected. Conditions in the human stomach differ from those in the murine stomach, including differences in terms of acidity^64,65^ anatomy^80^ and the availability of nutrients from food intake. This suggests that *H. pylori* might face distinctive selective forces in the human host compared to the mouse model resulting in differently adapted isolates. Moreover, by analyzing re-isolates, only long-term adaptations can be detected, potentially overlooking short-term changes in protein expression that might occur in a complex and changeable environment such as the stomach.

In summary, *H. pylori* gGT has a pivotal role in acid resistance, nitrogen metabolism and iron acquisition, while its loss induces several compensatory mechanisms to ensure bacterial colonization and persistence in the host. These findings may have potential implications for drug development. On the one hand, *H. pylori* gGT is a promising target for the development of new therapies, because the enzyme is functionally highly important for the bacterium on different levels such as immune evasion, acid resistance and nutritional acquisition. On the other hand, our results suggest, that targeting *H. pylori* gGT alone may not be sufficient to limit bacterial colonization, as *H. pylori* can compensate for the loss of the enzyme. Therefore, the combination with other targets identified in our study as potential compensatory candidates might mitigate the emergence of escape mutants.

## Supplementary Material

242466660R2_supplement figures.docx

## Data Availability

All data generated or analyzed during this study are included in this article. Mass spectrometry proteomics data have been deposited to the ProteomeXchange^81^ Consortium via the PRIDE^82^ partner repository with the dataset identifier PXD050334. Other datasets are available from the corresponding author on reasonable request.

## Notes

A pre-print of this manuscript has been published on bioRxiv.^83^
