# Supplementary material for: Helicobacter pylori γ-glutamyltransferase is linked to proteomic adaptions important for colonization
Source: Gut Microbes. 2025 Apr 9;17(1):2488048. doi: 10.1080/19490976.2025.2488048 (PMC11988274; doi:10.1080/19490976.2025.2488048)
Supplement: 242466660R2_supplement figures.docx [file KGMI_A_2488048_SM1514.docx]

**Supplementary Data**

**Supplementary Methods**

**Construction of conditional *H. pylori* gGT mutants**

Conditional gGT mutants were constructed using a counter selection system based on a *rpsL-cat* cassette as described by Dailidiene et al. and Debowski et al.^1, 2^ First, streptomycin resistant *H. pylori* strains were generated by replacing *H. pylori* *rpsL* by a mutated version. Then, the *ggt* gene was placed under the control of the *tet*-inducible promoter *tetO1* (P_tetO1_)^3^ and finally, *tetR* was introduced into the *H. pylori* genome close to the *mdaB* locus.

To exchange the *ggt* promoter plasmids pGGT_rpsL-cat and pGGT_tetO1 were constructed using a commercially available Gibson Assembly Cloning Kit (NEB). Homologous regions were amplified from the genomic DNA of strain PMSS1 using primer pairs FlgK fwd/FlgK rev (rpsL/tetO1) and ggt fwd (rpsL/tetO1)/ggt rev. The *rpsL-cat* cassette and the *tetO1* promoter were amplified from pOND708 and pOND817^3^ by the primers rpsL fwd/rev and tetO1 fwd/rev, respectively. pOND708 was opened by PCR using the primers backbone (rpsL/tetO1) fwd/rev and was used as a plasmid backbone. An overview of the cloning procedure (**Figure S1**, **A**), primers (**Table S2**) and plasmids (**Table S3**) is presented in the supplemental material.

Plasmids were naturally transformed to streptomycin-resistant *H. pylori* to replace the *ggt* promoter with *tetO1*. Transformants were tested for correct genomic insertions by PCR using the primers FlgK fwd seq and tetO1 rev. Next, natural transformation of pOND708 and pOND1162^3^ resulted in the introduction of *tetR* into the *H. pylori* genome. Correct insertion was verified by PCR using the primer pair TetR check fwd/rev.

**Secretome analysis**

Bacteria were resuspended in McIlvaine buffer (pH 5; 10^9^ bacteria/ml) and incubated for 2 h at 37 °C in microaerobic conditions before the supernatant was separated by centrifugation (5000 g, 10 min, 4 °C), filtered (PVDF, 0.22 μm) and PMSF (100 µM) was added to prevent protein degradation. Samples were prepared for MS analysis following a similar protocol as developed by Wisniewski et al using 30 kDa centrifugal units^4^. Filters were equilibrated with 500 µl 0.05 M NaOH (14 000 *g*, 15 min) and washed with UA buffer (8 M urea. 0.1 M Tris-HCl, pH 8.5) (14 000 *g*, 15 min) before use. Samples were reduced with DTT (1 mM) in UA buffer for 1 h at 37 °C. Filters were washed with UA (14 000 *g*, 15 min) and samples were alkylated by adding 100 µl of 50 mM IAA in UA, followed by mixing for 1 min at 600 rpm and 20 min incubation at room temperature. Filters were centrifuged (14 000 *g*, 10 min) and washed with 100 µl UA buffer (1x) and ABC buffer (0.05 M NH_4_HCO_3_) (2x). Protein digestion was done in 40 μl ABC buffer with trypsin (in 50 mM acetic acid, Promega*)* for 16 h at 37 °C in a wet chamber. Peptides were eluted in LoBind tubes (Eppendorf, 14 000 *g*, 10 min) followed by a wash step with 40 µl of ABC buffer. Peptide eluates were acidified with formic acid (pH < 6) and desalted before MS analysis was performed.

**Western blot**

*H. pylori* was lysed in RIPA buffer (50 mM Tris, 150 mM NaCl, 1 mM EGTA, 1% Igepal, 0.25% sodium deoxycholate, pH 7.4) by sonication for 10 min and protein concentration was measured using a commercial BCA-Assay Kit (ThermoFisher Scientific). Equal amounts of proteins were separated by SDS-PAGE and transferred by semi-dry blotting to a nitrocellulose membrane (Amersham Protran 0.45, GE Healthcare, Chicago, IL, USA). Blocking was performed in 5% (w/v) non-fat milk dissolved in TBS-T (Tris-buffered saline with 0.1% Tween 20 (*v*/*v*)) for one hour at room temperature. Primary antibody incubation was done overnight at 4 °C with antibodies targeting the *H. pylori* virulence factors urease B (ThermoFisher Scientific, 1:5000), CagA (1:3000), HpaA (1:5000) and gGT (1:500). Antibodies against HpaA and gGT were produced in-house in mice and anti-CagA was retrieved from rabbits.

After washing, an HRP-coupled secondary anti-rabbit or anti-mouse antibody was incubated with the membrane (Promega, 1:3000). For detection, Pierce ECL Western Blotting Substrate (ThermoFisher Scientific) was applied. Chemiluminescence was detected using the ChemoCam Imager ECL (Intas Science Imaging).

**Amidase and Formamidase Activity Assays**

Enzymatic activities of AmiE and AmiF were measured as previously described with some modifications ^5^**.** The assay protocol is identical to the urease activity assay in this study except for the reaction time (30 min), temperature (30°C), and the reaction medium (100 mM acrylamide or 1 mM formamide in PEB).

**Histological evaluation**

A longitudinal stomach section from the esophagus to the stomach antrum was fixed in 4% paraformaldehyde and embedded in paraffin. Tissue sections were evaluated after chloroacetate esterase staining (CAE) and immunohistochemistry (IHC). For IHC, antigen retrieval was achieved by boiling samples in 0.01 M sodium citrate at pH 6. Blocking was performed for 1 h in 5% goat serum. Primary antibodies (CD3: SP7, ThermoFisher Scientific; CD4: ab183685CD8, abcam; CD8: 49M15, ebioscience) were applied overnight at 4 °C. Detection was done by applying horse radish peroxidase-conjugated secondary antibodies (anti-rabbit HRP-conjugated, Promega; anti-rat HRP-conjugated, Dako) for 1 h at room temperature and by subsequent color development using the SignalStain DAB substrate kit (Cell Signaling) according to the manufacturer’s instructions. Images were acquired using the Aperio AT2 system (Leica).

**Supplementary Tables**

**Table S1: Strains used in this study**

| **Strain name** | **Description** | **Reference** | **Antibiotic selection** |
| --- | --- | --- | --- |
| G27 wt | Wild type strain | ^6^ | - |
| PMSS1 wt | Wild type strain | ^7^ | - |
| G27 Δ*ggt* | Kan^R^ within the *ggt* gene | ^8^ | Kanamycin |
| PMSS1 Δ*ggt* | Kan^R^ within the *ggt* gene | ^9^ | Kanamycin |
| G27 Δ*ureAB* | Kan^R^ within the *ureA/B* gene | ^10^ | Kanamycin |
| PMSS1 Δ*ureAB* | Kan^R^ within the *ureA/B* gene | This work | Kanamycin |
| PMSS1 strep^+^ | Mutated *H. pylori rpsL* | This work | Streptomycin |
| G27 strep^+^ | Mutated *H. pylori rpsL* | This work | Streptomycin |
| PMSS1 *ggt::rpsL-cat* | *rpsL-cat* instead of the *ggt*-promoter | This work | Chloramphenicol |
| PMSS1 *ggt::tetO1*  Other name:  PMSS1 *g::O1 C* | *tetO1* instead of the *ggt*-promoter | This work | Streptomycin |
| PMSS1 *mdaB::rpsL-cat* | *tetO1* instead of the *ggt*-promoter and *rpsL-cat* next to the *mdaB* locus | This work | Chloramphenicol |
| PMSS1 *ggt::tetO1 tetR*  Other name:  PMSS1 *g::O1 t* | tet-on gGT-strain, *tetO1* instead of the *ggt*-promoter and *tetR* inserted next to the *mdaB* locus | This work | Streptomycin |
| G27 *mdaB:: rpsL-cat* | *tetO1* instead of the *ggt*-promoter and *rpsL-cat* next to the *mdaB* locus | This work | Chloramphenicol |
| G27 *mdaB::tetR* | *tetR* inserted next to the *mdaB* locus | This work | Streptomycin |
| G27 *ggt::rpsL-cat* | *rpsL-cat* instead of the *ggt*-promoter, *tetR* inserted next to the *mdaB* locus | This work | Chloramphenicol |
| G27 *ggt::tetO1 tetR*  Other name:  G27 *g::O1 t* | tet-on gGT-strain, *tetO1* instead of the *ggt*-promoter and *tetR* inserted next to the *mdaB* locus | This work | Streptomycin |

**Table S2: Oligonucleotide primers used in this study**

| **Primer Name** | **Primer Sequence** | **Use** |
| --- | --- | --- |
| **FlgK fwd** | **cggtatcgat**TCGCTGAGATCAACAAACGC | **Amplification of components for Gibson Assembly of plasmid  pGGT_rpsL-cat** |
| **FlgK rev (rpsL)** | **catagttata**GAGCGCTGGCTATAAAAAAG |  |
| **rpsL fwd** | **gccagcgctc**TATAACTATGGATTAAACACTTTTTTAG |  |
| **rpsL rev** | **cgcataacca**TTCAGCAAGTCTTGTAATTC |  |
| **ggt flank fwd (rpsL)** | **acttgctgaa**TGGTTATGCGATTTCACAAAG |  |
| **ggt flank rev (rpsL)** | **tggcggccgc**TAGTGAGGTTGTCTTTCACATC |  |
| **backbone fwd (rpsL)** | **aacctcacta**GCGGCCGCCACCGCGGTGGA |  |
| **backbone rev** | **atctcagcga**ATCGATACCGTCGATCGAGGGGGGGCCC |  |
| **FlgK fwd** | see above | **Amplification of components for Gibson Assembly of plasmid pGGT_tetO1** |
| **FlgK rev (tetO1)** | **tagaagatct**GAGCGCTGGCTATAAAAAAG |  |
| **tetO1 fwd** | **gccagcgctc**AGATCTTCTAGAGATTAGTTAATG |  |
| **tetO1 rev** | **tccgtctcat**ATGTTATTCTCCTATTCTTAAAGTG |  |
| **ggt fwd (tetO1)** | **agaataacat**ATGAGACGGAGTTTTTTAAAAACG |  |
| **ggt rev** | **tggcggccgc**TCACTTGCAACACCGTAGTG |  |
| **backbone fwd (tetO1)** | **ttgcaagtga**GCGGCCGCCACCGCGGTGGA |  |
| **backbone rev** | see above |  |
| **TetR seq fwd** | ATCCTGGCAGTCATTTCTGCG | **Verification of the insertion of *tetR* next to the mdaB locus** |
| **TetR seq rev** | TGCTCACCATCATCACTGTCC |  |
| **Flgk seq fwd** | AGAGCCTACCACTATCCGC | **Verification of the insertion of *tetO1* instead of the *ggt* promotor** |
| **tetO1 rev** | see above |  |
| **ggt fwd**  **ggt rev** | ATTCGCATCGCCCCCTACTA  GAAGCGATGCGTCAGGCTTA | **Verification of the deletion of *ggt*** |
| **ureAB fwd**  **ureAB rev** | ATGAAACTCACCCCAAAAGAGTT  TTACTCCTTAATTGTTTTTACATAGTTG | **Verification of the deletion of *ureAB*** |

**Bold letters:** overhang for Gibson Assembly**,** BIG LETTERS: annealing part of the primer

**Table S3: Plasmids used in this study**

| **Plasmid name** | **description** | **reference** |
| --- | --- | --- |
| **pOND708** | derivative of pBlu-SK-alt with homologous regions to *hydA* and *mdaB* flanking *rpsL-cat* | ^3^ |
| **pOND817** | derivative of pBlu-SK-alt with homologous region to *trpB/A* flanking P_tetO1_-GFP | ^3^ |
| **pOND1162** | derivative of pBlu-SK-alt with homologous regions to *hydA* and *mdaB* flanking P_flaA_-tetR | ^3^ |
| **pGGT_rpsL-cat** | derivative of pOND708 with homologous regions to the upstream and downstream DNA sequence of the *ggt*-promoter flanking *rpsL-cat* | This work |
| **pGGT_tetO1** | derivative of pOND708 with homologous regions to the upstream and downstream DNA sequence of the *ggt*-promoter flanking *tetO1* | This work |

**Table S4:** **Significantly different proteins comparing the proteome of PMSS1 wt re-isolates with PMSS1 ∆ggt re-isolates**

| **Protein IDs** | **Fasta headers** | **-Log_10_**  **(p-value)** | **Log_2_**  **(fold change)** | **Time of infection** |
| --- | --- | --- | --- | --- |
| A0A3T1CRE4 | Site-specific DNA-methyltransferase (adenine-specific) | 7.7 | 3.9 | **1 week** |
| A0A3T1CR86 | Type III restriction enzyme, res subunit | 4.2 | 3.0 |  |
| A0A3Q9Y164 | Oligopeptide-binding protein **OppA** | 7.3 | 2.6 |  |
| A0A3T1CRZ1 | Bacterial extracellular solute-binding proteins, family 5 Middle | 11.5 | 2.4 |  |
| A0A3T1CR68 | Restriction endonulease | 2.1 | 1.1 |  |
| A0A3T1CUZ9 | L-serine dehydratase **SdaA** | 7.4 | 2.1 |  |
| A0A3Q9XZ75 | **RloF** | 1.8 | 1.9 |  |
| A0A3T1CVF8 | Fe(3+) dicitrate transport protein FecAprecursor **FecA3** | 5.9 | 1.2 |  |
| A0A3Q9Y2Z1 | Uncharacterized protein | 1.6 | -1.0 |  |
| A0A0B2EX11 | Flagellar assembly factor **FliW** | 1.9 | -1.1 |  |
| Q7X3Z4 | Helicobacter outer membrane protein | 3.2 | -1.1 |  |
| A0A2R4LB11 | Flagellar biosynthesis protein **FlaG** | 1.8 | -1.2 |  |
| A0A3T1CTE4 | Flagellar hook-length control protein **FliK** | 3.2 | -2.0 |  |
| A0A0B2E5J3 | Putative lipoprotein | 2.7 | -2.8 |  |
| A0A3Q9Y098 | Uncharacterized protein | 8.0 | -3.7 |  |
| A0A3Q9XTR1 | Neuraminyllactose-binding hemagglutinin **HpaA** | 10.9 | -5.8 |  |
| A0A3Q9Y1A7 | Glutathione hydrolase proenzyme **gGT** | 11.7 | -9.7 |  |
|  |  |  |  |  |
| A0A3T1CRE4 | Site-specific DNA-methyltransferase (adenine-specific) | 7.4 | 3.5 | **1 month** |
| A0A3T1CR86 | Type III restriction enzyme, res subunit | 4.3 | 2.9 |  |
| A0A3T1CVF8 | Fe(3+) dicitrate transport protein FecAprecursor **FecA3** | 7.2 | 2.4 |  |
| A0A3T1CUZ9 | L-serine dehydratase **SdaA** | 4.8 | 1.8 |  |
| A0A3Q9Y1S4 | Outer membrane beta-barrel protein **HofC** | 4.7 | 1.5 |  |
| A0A3Q9Y164 | Oligopeptide-binding protein **OppA** | 3.5 | 1.3 |  |
| A0A2T6SAQ1 | Diacylglycerol kinase | 1.6 | 1.2 |  |
| A0A3T1CU26 | Helicobacter outer membrane protein **HopJ/K** | 2.6 | 1.2 |  |
| A0A3Q9Y1U5 | Putative outer membrane protein **HofB** | 3.7 | 1.1 |  |
| A0A3T1CR68 | Restriction endonulease | 2.1 | 1.1 |  |
| A0A438WM48 | Uncharacterized protein | 5.7 | 1.0 |  |
| A0A0B2E9E8 | ABC transporter permease **DppB** | 1.8 | -1.1 |  |
| A0A2A6S9G2 | ABC transporter ATP-binding protein **OppF** | 1.9 | -1.2 |  |
| Q6VRL4 | Cag pathogenicity island protein | 1.6 | -1.4 |  |
| A0A3Q9Y098 | Uncharacterized protein | 3.3 | -1.6 |  |
| A0A3Q9XZX7 | DUF262 domain-containing protein | 1.5 | -1.7 |  |
| A0A3Q9XTR1 | Neuraminyllactose-binding hemagglutinin **HpaA** | 8.2 | -6.4 |  |
| A0A3Q9Y1A7 | Glutathione hydrolase proenzyme **gGT** | 10.1 | -9.6 |  |
|  |  |  |  |  |
| A0A3Q9Y0Y8 | Heme transporter **FrpB1** | 2.9 | 2.8 | **3 months** |
| A0A3Q9XTN3 | Uncharacterized protein | 5.1 | 2.1 |  |
| A0A438Q960 | Helicobacter outer membrane protein | 2.8 | 2.1 |  |
| A0A3Q9Y1S4 | Outer membrane beta-barrel protein **HofC** | 4.5 | 2.0 |  |
| A0A0B2E5F6 | Fe(3+) dicitrate transport protein FecAprecursor **FecA1** | 1.9 | 1.8 |  |
| A0A3T1CVF8 | Fe(3+) dicitrate transport protein FecAprecursor **FecA3** | 1.5 | 1.8 |  |
| A0A3Q9Y0C6 | Helicobacter outer membrane protein **HopE** | 4.0 | 1.7 |  |
| A0A3Q9Y1U5 | Putative outer membrane protein **HofB** | 4.7 | 1.7 |  |
| A0A3Q9Y0T3 | TonB-dependent heme receptor A **TdhA** | 2.3 | 1.7 |  |
| A0A3T1CUZ9 | L-serine dehydratase **SdaA** | 4.4 | 1.7 |  |
|  |  |  |  |  |
| A0A3Q9XYL3 | Outer membrane efflux protein | 5.2 | 1.5 |  |
| A0A3T1CTF9 | Helicobacter outer membrane protein **AlpA** | 5.2 | 1.5 |  |
| A0A3Q9Y0G6 | Heme transporter **FrpB2** | 1.7 | 1.2 |  |
| A0A024BZ45 | Helicobacter outer membrane protein (**HorB**) | 2.0 | 1.2 |  |
| A0A3Q9Y164 | Oligopeptide-binding protein **OppA** | 1.5 | 1.1 |  |
| A0A3T1CUK3 | Helicobacter outer membrane protein | 2.7 | 1.1 |  |
| A0A3Q9Y015 | Uncharacterized protein | 6.8 | 1.1 |  |
| A0A3T1CR68 | Restriction endonulease | 2.1 | 1.1 |  |
| A0A3Q9Y348 | Helicobacter outer membrane protein | 4.9 | 1.0 |  |
| A0A3Q9XZW6 | Helicobacter outer membrane protein | 3.2 | 1.0 |  |
| A0A3T1CS29 | Helicobacter pylori **IceA2** protein | 1.8 | -1.1 |  |
| A0A3T1CV56 | ATP-dependent Clp protease ATP-binding subunit **ClpA** | 3.0 | -1.1 |  |
| A0A438RXP5 | Protoporphyrinogen IX oxidase | 1.3 | -1.3 |  |
| A0A2A6W2V4 | Chorismate mutase | 2.4 | -1.3 |  |
| A0A3T1CS89 | Endoribonuclease **YbeY** | 2.2 | -1.5 |  |
| A0A024BXZ2 | 50S ribosomal protein L35 | 4.1 | -1.6 |  |
| A0A3Q9Y098 | Uncharacterized protein | 1.9 | -1.7 |  |
| A0A3Q9XTR1 | Neuraminyllactose-binding hemagglutinin **HpaA** | 5.5 | -7.4 |  |
| A0A3Q9Y1A7 | Glutathione hydrolase proenzyme **gGT** | 9.5 | -9.6 |  |

**Table S5: Significantly different proteins comparing the proteome of PMSS1 wt with PMSS1 ∆ggt**

| **Protein IDs** | **Fasta headers** | **-Log10**  **(p-value)** | **Log2 (fold change)** | **Experi-ment** |
| --- | --- | --- | --- | --- |
| A0A3Q9XZY6 | Type III restriction enzyme, res subunit | 3.5 | 2.3 | **I** |
| A0A3Q9XZ75 | **RloF** | 2.6 | 1.9 |  |
| A0A3Q9XTR1 | Neuraminyllactose-binding hemagglutinin **HpaA** | 3.7 | 1.9 |  |
| A0A0B2DY50 | Formamidase **AmiF** | 2.8 | 1.6 |  |
| A0A3T1CS01 | Helicobacter outer membrane protein | 1.8 | 1.6 |  |
| A0A3T1CUZ9 | L-serine dehydratase **SdaA** | 2.7 | 1.4 |  |
| A0A3T1CRZ1 | Bacterial extracellular solute-binding proteins, family 5 Middle | 1.6 | 1.4 |  |
| A0A3T1CRE4 | Site-specific DNA-methyltransferase (adenine-specific) | 2.0 | 1.4 |  |
| A0A024C5N3 | Lipoprotein | 3.6 | 1.1 |  |
| A0A3Q9Y151 | Motility accessory factor | 2.7 | -1.0 |  |
| A0A2T6SBW1 | Uncharacterized protein | 1.4 | -1.1 |  |
| Q7X3Z4 | Helicobacter outer membrane protein, **HorE** | 1.4 | -1.1 |  |
| A0A0B2EX11 | Flagellar assembly factor **FliW** | 1.8 | -1.1 |  |
| A0A3Q9Y110 | Uncharacterized protein | 2.1 | -1.2 |  |
| A0A3Q9Y238 | Phosphoethanolamine transferase **CptA** | 4.5 | -2.5 |  |
| A0A3Q9Y098 | Uncharacterized protein | 8.0 | -3.7 |  |
| A0A3Q9Y1A7 | Glutathione hydrolase proenzyme **gGT** | 3.0 | -8.8 |  |
|  |  |  |  |  |
| A0A3T1CRF2 | Uncharacterized protein | 3.1 | 3.5 | **II** |
| A0A3T1CRE4 | Site-specific DNA-methyltransferase (adenine-specific) | 3.2 | 3.0 |  |
| A0A3T1CR86 | Type III restriction enzyme, res subunit | 2.5 | 2.8 |  |
| A0A3Q9XZ75 | Uncharacterized protein **RloF** | 1.8 | 2.6 |  |
| A0A024C5N3 | Lipoprotein | 3.5 | 2.2 |  |
| A0A3Q9XTR1 | Neuraminyllactose-binding hemagglutinin **HpaA** | 2.3 | 2.1 |  |
| A0A3T1CVF8 | Fe(3+) dicitrate transport protein FecAprecursor, **FecA3** | 2.8 | 1.9 |  |
| A0A0B2DY50 | Formamidase **AmiF** | 2.6 | 1.6 |  |
| A0A3T1CUZ9 | L-serine dehydratase **SdaA** | 3.1 | 1.4 |  |
| A0A0B2E752 | Acetone carboxylase alpha subunit, **AcxB** | 3.0 | 1.4 |  |
| A0A3Q9Y0G4 | N-glycosylase/DNA lyase | 1.3 | 1.3 |  |
| A0A3Q9XZK4 | Lipoprotein | 1.4 | 1.2 |  |
| A0A438WM48 | Uncharacterized protein | 1.4 | 1.1 |  |
| A0A3T1CS96 | Outer membrane protein P6 | 1.5 | 1.0 |  |
| Q7X3Z4 | Helicobacter outer membrane protein, **Omp11** | 1.4 | -1.1 |  |
| A0A3Q9Y238 | Phosphoethanolamine transferase **CptA** | 2.4 | -1.1 |  |
| A0A3T1CTI6 | RNA polymerase sigma-54 factor | 2.3 | -1.1 |  |
| A0A3T1CUE6 | Putative pre-16S rRNA nuclease | 1.9 | -1.3 |  |
| A0A2A6UTA0 | Flagellin **FlaA** | 2.3 | -1.3 |  |
| A0A3Q9Y151 | Motility accessory factor | 3.1 | -1.3 |  |
| Q6VYQ1 | Flagellin **FlaB** | 2.3 | -1.4 |  |
| A0A3T1CSE3 | Cytosine-specific methyltransferase | 1.5 | -1.7 |  |
| A0A024C2H7 | ATP synthase subunit c | 1.7 | -1.7 |  |
| A0A0B2EX11 | Flagellar assembly factor **FliW** | 2.4 | -2.1 |  |
| A0A3Q9Y098 | Uncharacterized protein | 8.0 | -3.7 |  |
| A0A3T1CS01 | Helicobacter outer membrane protein | 3.0 | -2.8 |  |
| A0A3Q9Y1A7 | Glutathione hydrolase proenzyme **gGT** | 5.1 | -8.8 |  |
|  |  |  |  |  |
|  |  |  |  |  |
| A0A3T1CTK4 | Modulator of FtsH protease YccA | 2.8 | 2.8 | **III** |
| A0A3T1CR86 | Type III restriction enzyme, res subunit | 1.9 | 2.6 |  |
| A0A3T1CVF8 | Fe(3+) dicitrate transport protein FecAprecursor **FecA3** | 1.6 | 2.6 |  |
| A0A3T1CRE4 | Site-specific DNA-methyltransferase (adenine-specific) | 1.9 | 2.4 |  |
| A0A0B2DY50 | Formamidase **AmiF** | 1.5 | 2.2 |  |
| A0A3Q9Y0Y8 | Heme transporter **BhuA** | 1.5 | 1.8 |  |
| A0A3Q9XXS3 | 4-diphosphocytidyl-2-C-methyl-D-erythritol kinase | 1.8 | 1.7 |  |
| A0A1V3ARV0 | Cb-type cytochrome c oxidase subunit IV | 1.7 | 1.7 |  |
| A0A3Q9XTN3 | Uncharacterized protein | 6.7 | 1.5 |  |
| A0A024C5N3 | Lipoprotein | 2.9 | 1.4 |  |
| A0A3T1CUZ9 | L-serine dehydratase **SdaA** | 2.9 | 1.3 |  |
| A0A3Q9XZQ1 | Site-specific DNA-methyltransferase (adenine-specific) | 3.1 | 1.2 |  |
| A0A3T1CR68 | Uncharacterized protein | 1.2 | 1.2 |  |
| A0A3Q9Y373 | Uncharacterized protein | 2.6 | 1.2 |  |
| A0A3T1CSQ0 | Adenosyl-chloride synthase | 1.4 | 1.2 |  |
| A0A3T1CU26 | Helicobacter outer membrane protein | 1.5 | 1.1 |  |
| A0A3T1CV56 | ATP-dependent Clp protease ATP-binding subunit **ClpA** | 2.2 | -1.0 |  |
| Q6VYQ1 | Flagellin **FlaB** | 2.3 | -1.0 |  |
| A0A2R4LB11 | Flagellar biosynthesis protein **FlaG** | 3.0 | -1.1 |  |
| A0A2T6SC88 | R.HinP1I restriction endonuclease | 2.6 | -1.1 |  |
| Q7X3Z4 | Helicobacter outer membrane protein **Omp11** | 2.9 | -1.2 |  |
| A0A3Q9Y0I8 | Flagellar hook-associated protein 2 **FliD** | 2.4 | -1.2 |  |
| A0A1W0VN70 | NADH-quinone oxidoreductase subunit J | 2.5 | -1.3 |  |
| A0A3Q9Y151 | Motility accessory factor | 5.1 | -1.4 |  |
| A0A3Q9Y134 | Uncharacterized protein | 1.5 | -1.4 |  |
| A0A3Q9Y383 | Uncharacterized protein | 1.4 | -1.4 |  |
| A0A3Q9Y238 | Phosphoethanolamine transferase **CptA** | 1.6 | -1.5 |  |
| A0A0B2EP32 | ABC transporter permease **DppC** | 1.4 | -1.8 |  |
| A0A3T1CSE3 | Cytosine-specific methyltransferase | 2.1 | -1.9 |  |
| A0A0B2EX11 | Flagellar assembly factor **FliW** | 1.3 | -2.4 |  |
| A0A3Q9Y098 | Uncharacterized protein | 8.0 | -3.7 |  |
| A0A3Q9Y1A7 | Glutathione hydrolase proenzyme **gGT** | 2.5 | -9.4 |  |

**Table S6: Significantly different proteins comparing the proteome of PMSS1 ∆ggt re-isolates with the proteome of the PMSS1 ∆ggt isolate used for infection**

| **Protein IDs** | **Fasta headers** | **-Log10**  **(p-value)** | **Log2 (fold change)** | **Time of infection** |
| --- | --- | --- | --- | --- |
| A0A3Q9Y0T3 | TonB-dependent heme receptor A **TdhA** | 2.5 | 2.9 | **1 week** |
| A0A3Q9Y238 | Phosphoethanolamine transferase **CptA** | 7.5 | 2.7 |  |
| A0A3T1CVF8 | Fe(3+) dicitrate transport protein FecAprecursor **FecA3** | 6.9 | 2.3 |  |
| A0A3Q9Y1U5 | Putative outer membrane protein **HofB** | 6.9 | 2.3 |  |
| A0A3Q9Y1S4 | Outer membrane beta-barrel protein **HofC** | 6.6 | 2.3 |  |
| A0A0S1XUM1 | Outer membrane beta-barrel protein **HofG** | 7.3 | 2.1 |  |
| A0A3T1CRE4 | Site-specific DNA-methyltransferase (adenine-specific) | 3.8 | 2.0 |  |
| A0A3Q9Y0C6 | Helicobacter outer membrane protein **HopE** | 5.0 | 2.0 |  |
| A0A3T1CR68 | Restriction endonuclease | 5.5 | 1.9 |  |
| A0A3Q9Y0Y8 | Heme transporter **FrpB1** | 7.6 | 1.9 |  |
| A0A024BZ45 | Helicobacter outer membrane protein **HorB** | 3.8 | 1.5 |  |
| A0A438Q960 | Helicobacter outer membrane protein, **Omp14** | 4.0 | 1.4 |  |
| A0A3Q9Y0G6 | Heme transporter **FrpB2** | 2.8 | 1.4 |  |
| A0A3T1CRA7 | Helicobacter outer membrane protein **HopK** | 5.8 | 1.4 |  |
| A0A3T1CR86 | Type III restriction enzyme, res subunit | 1.8 | 1.4 |  |
| A0A3Q9XYL3 | Outer membrane efflux protein | 6.1 | 1.3 |  |
| A0A3Q9Y164 | Oligopeptide-binding protein **OppA** | 4.8 | 1.3 |  |
| A0A0B2E5F6 | Fe(3+) dicitrate transport protein FecAprecursor, **FecA1** | 6.1 | 1.2 |  |
| A0A3T1CU26 | Helicobacter outer membrane protein **HopJ/K** | 3.6 | 1.1 |  |
| A0A0B2E4K4 | 50S ribosomal protein **L18** | 5.5 | -1.0 |  |
| A0A3Q9XZQ1 | Site-specific DNA-methyltransferase (adenine-specific), **YhdJ** | 3.3 | -1.7 |  |
| A0A0B2E5J3 | Putative lipoprotein | 3.0 | -3.3 |  |
| A0A3Q9XZY6 | Type III restriction enzyme, res subunit | 5.8 | -4.5 |  |
| A0A3Q9XTR1 | Neuraminyllactose-binding hemagglutinin **HpaA** | 6.6 | -7.5 |  |
|  |  |  |  |  |
| A0A3T1CSE3 | Cytosine-specific methyltransferase | 3.0 | 1.8 | **1 month** |
| A0A3T1CTE2 | Uncharacterized protein | 2.4 | 1.7 |  |
| A0A0B2EX11 | Flagellar assembly factor **FliW** | 2.4 | 1.7 |  |
| A0A2A6UTA0 | Flagellin **FlaA** | 3.0 | 1.6 |  |
| Q6VYQ1 | Flagellin **FlaB** | 3.4 | 1.5 |  |
| A0A2T6SBW1 | Uncharacterized protein | 2.3 | 1.4 |  |
| Q7X3Z4 | Helicobacter outer membrane protein, **HorE** | 3.4 | 1.4 |  |
| A0A3T1CUE6 | Putative pre-16S rRNA nuclease | 5.4 | 1.4 |  |
| A0A3Q9Y238 | Phosphoethanolamine transferase **CptA** | 2.9 | 1.2 |  |
| A0A3Q9Y032 | RNA pyrophosphohydrolase **RppH** | 2.3 | 1.2 |  |
| A0A3Q9Y1S0 | Flagellar hook protein **FlgE** | 4.4 | 1.1 |  |
| A0A3T1CS89 | Endoribonuclease **YbeY** | 1.5 | 1.1 |  |
| A0A3Q9Y232 | Ribosomal RNA small subunit methyltransferase A, **RsmA** | 1.3 | 1.1 |  |
| A0A3Q9XYC9 | Modulator of drug activity B, **MdaB** | 1.8 | 1.0 |  |
| A0A3Q9XS18 | DNA polymerase III subunit delta | 1.8 | 1.0 |  |
| A0A3T1CTB2 | **CagZ** | 1.3 | -1.0 |  |
| A0A3T1CR94 | Uncharacterized protein | 2.9 | -1.2 |  |
| A0A3Q9Y1R6 | HaeIII restriction endonuclease | 1.6 | -1.2 |  |
| A0A0B2E9E8 | ABC transporter permease, **DppB** | 1.3 | -1.6 |  |
| A0A3Q9XZ75 | **RloF** | 1.9 | -2.0 |  |
| A0A3Q9XZX7 | DUF262 domain-containing protein | 2.8 | -2.1 |  |
| A0A024C5N3 | Lipoprotein | 4.9 | -2.1 |  |
| A0A3Q9XZY6 | Type III restriction enzyme, res subunit | 2.5 | -2.3 |  |
| A0A3T1CRF2 | Uncharacterized protein | 3.6 | -2.9 |  |
| A0A3Q9Y0T3 | TonB-dependent heme receptor A **TdhA** | 2.2 | -3.0 |  |
| A0A3Q9XZQ1 | Site-specific DNA-methyltransferase (adenine-specific), **YhdJ** | 1.9 | -7.1 |  |
| A0A3Q9XTR1 | Neuraminyllactose-binding hemagglutinin **HpaA** | 6.3 | -7.0 |  |
|  |  |  |  |  |
| A0A0B2EX11 | Flagellar assembly factor **FliW** | 2.1 | 2.7 | **3 months** |
| A0A3Q9XYG4 | L,D-transpeptidase catalytic domain | 1.7 | 1.6 |  |
| A0A3T1CSE3 | Cytosine-specific methyltransferase | 2.6 | 1.5 |  |
| Q6VYQ1 | Flagellin **FlaB** | 3.4 | 1.4 |  |
| A0A2A6UTA0 | Flagellin **FlaA** | 3.5 | 1.3 |  |
| A0A3Q9XYL3 | Outer membrane efflux protein | 3.6 | 1.2 |  |
| Q7X3Z4 | Helicobacter outer membrane protein**, HorE** | 4.9 | 1.2 |  |
| A0A3Q9XZK4 | Lipoprotein | 1.9 | 1.2 |  |
| A0A1W0VN70 | NADH-quinone oxidoreductase subunit J, **NuoJ** | 1.5 | 1.2 |  |
| A0A3T1CTH1 | DNA helicase, **UvrD** | 1.5 | 1.1 |  |
| A0A0S1XUM1 | Outer membrane beta-barrel protein **HofG** | 1.9 | 1.1 |  |
| A0A2A6YFN3 | Carboxy-terminal processing protease **CtpB** | 4.9 | 1.1 |  |
| A0A3Q9Y151 | Motility accessory factor | 4.9 | 1.1 |  |
| A0A3Q9Y1S0 | Flagellar hook protein **FlgE** | 3.4 | 1.0 |  |
| A0A3Q9Y1S4 | Outer membrane beta-barrel protein **HofC** | 2.2 | 1.0 |  |
| A0A2R4LB11 | Flagellar biosynthesis protein **FlaG** | 4.7 | 1.0 |  |
| A0A024C5N3 | Lipoprotein | 3.1 | -1.2 |  |
| A0A3T1CS89 | Endoribonuclease **YbeY** | 1.5 | -2.6 |  |
| A0A3Q9XZY6 | Type III restriction enzyme, res subunit | 1.4 | -2.0 |  |
| A0A0B2DY50 | Formamidase **AmiF** | 2.6 | -2.3 |  |
| A0A3Q9XTR1 | Neuraminyllactose-binding hemagglutinin **HpaA** | 4.3 | -6.3 |  |

**Table S7: Significantly different proteins comparing the proteome of PMSS1 wt re-isolates with the proteome of the PMSS1 wt isolate used for infection**

| **Protein IDs** | **Fasta headers** | **-Log10(p-value)** | **Log2 (fold change)** | **Time of infection** |
| --- | --- | --- | --- | --- |
| A0A0S1XUM1 | Outer membrane beta-barrel protein **HofG** | 3.4 | 2.2 | **1 week** |
| A0A024C2H7 | ATP synthase subunit c | 2.8 | 1.9 |  |
| A0A3Q9Y0T3 | TonB-dependent heme receptor A **TdhA** | 1.9 | 1.8 |  |
| A0A3Q9Y1U5 | Putative outer membrane protein **HofB** | 3.7 | 1.7 |  |
| A0A3Q9Y1S4 | Outer membrane beta-barrel protein **HofC** | 4.7 | 1.5 |  |
| A0A024C8K2 | Biopolymer transport protein **ExbB** | 1.7 | 1.5 |  |
| A0A3T1CVF8 | Fe(3+) dicitrate transport protein FecAprecursor **FecA3** | 3.6 | 1.4 |  |
| A0A3Q9Y0Y8 | Heme transporter **BhuA** | 4.2 | 1.2 |  |
| A0A3Q9XYL3 | Outer membrane efflux protein | 4.5 | 1.1 |  |
| A0A3T1CU26 | Helicobacter outer membrane protein **HopJ/K** | 3.7 | 1.1 |  |
| A0A3Q9Y0C6 | Helicobacter outer membrane protein **HopE** | 3.6 | 1.1 |  |
| A0A3Q9XZQ1 | Site-specific DNA-methyltransferase (adenine-specific) **YhdJ** | 4.5 | -2.8 |  |
| A0A3Q9XZY6 | Type III restriction enzyme, res subunit | 4.2 | -2.9 |  |
|  |  |  |  |  |
| A0A0B2E6I9 | Uncharacterized protein | 1.5 | 1.8 | **1 month** |
| A0A3Q9XYC9 | Modulator of drug activity B, **MdaB** | 1.4 | 1.6 |  |
| A0A3T1CT80 | **CagU** | 2.1 | 1.5 |  |
| A0A024C9K1 | DUF904 domain-containing protein | 1.6 | 1.4 |  |
| A0A0B2DYB6 | DUF465 domain-containing protein | 1.9 | 1.4 |  |
| A0A293TQR4 | NADPH-dependent 7-cyano-7-deazaguanine reductase, **QueF** | 1.7 | 1.4 |  |
| A0A3T1CV57 | Methyltransferase, **DpnA5** | 1.2 | 1.3 |  |
| A0A2T6SBW1 | Uncharacterized protein | 1.8 | 1.2 |  |
| Q6YFK6 | Inorganic pyrophosphatase, **PPa** | 1.3 | 1.2 |  |
| A0A083YEV7 | Nucleoid-associated protein AA976_00460 | 1.4 | 1.1 |  |
| A0A3T1CTE4 | Flagellar hook-length control protein **FliK** | 1.6 | 1.0 |  |
| A0A3T1CU83 | Universal bacterial protein **YeaZ** | 1.4 | -1.1 |  |
| A0A2T6VY13 | Ribonuclease P protein component, **RnpA** | 1.4 | -1.3 |  |
| A0A024C2H7 | ATP synthase subunit c, **ATpE** | 2.0 | -1.6 |  |
| A0A3Q9XZY6 | Type III restriction enzyme, res subunit | 4.4 | -2.3 |  |
| A0A3T1CS01 | Helicobacter outer membrane protein | 3.1 | -2.3 |  |
|  |  |  |  |  |
| A0A3T1CTK4 | Modulator of FtsH protease **YccA** | 2.4 | 2.5 | **3 months** |
| A0A3Q9XXS3 | 4-diphosphocytidyl-2-C-methyl-D-erythritol kinase, **IspE** | 1.9 | 1.4 |  |
| A0A3Q9XYU7 | Protein-export membrane protein **SecG** | 1.9 | 1.3 |  |
| A0A1W0VN70 | NADH-quinone oxidoreductase subunit J, **NuoJ** | 1.3 | -1.1 |  |
| A0A3Q9XZY6 | Type III restriction enzyme, res subunit | 1.9 | -2.8 |  |
| A0A3Q9Y098 | Uncharacterized protein | 1.3 | -1.8 |  |

**Table S8: Differences between PMSS1 wt and PMSS1 ∆ggt detected by whole genome sequencing**

| **PMSS1**  **Locus** | **Gene** | **putative function** | **Uniprot Identifier** | **Difference *wt:∆ggt*** | **effect** |
| --- | --- | --- | --- | --- | --- |
| HPYL  PMSS1_  00113 |  | DUF874 containing protein | A0AAN1AVK5 | 7 single base pair mutations at end of the protein; position 858 C->A; 892 A-> G; 896 A-> G; 924 T-> C; 927 A-> G; 930 G->A; 935 A->T | 3 amino acid exchanges 298 I->V; 299 N->S; 312 A ->V; mutated in all PMSS1 ∆ggt isolates |
| HPYL  PMSS1_  00123 | *sdaA* | L-serine dehydratase SdaA | A0A3T1CUZ9 | 226 T-> C, T creates a stop codon in wt strains,  no stop codon with base pair exchange | disrupted gene in all wt strains,  full length gene in all ∆*ggt* re-isolates |
| HPYL  PMSS1_  00278 | *imaA* | autotransporter ImaA | A0AAN1AVS6 | single base pair mutation, 4375 T->C | 4377 S-> P; mutations in all PMSS1 ∆ggt isolates  + 3M_wt_RI_II |
| HPYL  PMSS1_  00983 | *hpaA* | Neuraminyllactose-binding  hemagglutinin HpaA | A0A3Q9XTR1 | wt BI: stop codon at position 31  ∆*ggt* BI: no stop codon because 91 C->T single base pair mutation  wt RIs: restoration of full length gene in two 3 M re-isolates ∆*ggt* RIs: different frameshift mutations in all re-isolates; no full length protein | wt BI: disrupted gene  ∆*ggt* BI: full length gene  wt RIs: disrupted or full length gene ∆*ggt* RIs: disrupted gene |
| HPYL  PMSS1_  01060 |  | secretory IgA binding protein EsiB;  putative beta-lactamase | A0AAN1AX83 | 13 C-> A, 41 T->C; 48 G->A, 54 G->A, 61 A->G, 79 T->C;  144 A-> G; 165 T->C, 186 G->A, 196 G->A; 234 G-> A;  342 T-> G; 357 A->G; 369 A-> G, 387 A-> C, 393 A-> G;  408 T-> C, 447 C-> A; 485 A->G; 546 T->C; 558 G-> A; 630 A->G; 651 G-> A, 688 C->A | 485 K->G; 196 V-> I, 79 F->L;  61 T->A, 41 V->A, 13 R->S;  mutations in all PMSS1 ∆*ggt* isolates |
| HPYL  PMSS1_  01061 | *ggt* | Gamma glutamyltransferase gGT | A0A3Q9Y1A7 | insertion of an aminoglycoside 3'-phosphotransferase gene   (kanamycin resistance) in all ∆*ggt* isolates | deletion of gGT in all ∆*ggt* isolates |
| HPYL  PMSS1_  01201 | *oppA* | Oligopeptide-binding protein  OppA | A0A3Q9Y164 | gene is disrupted in wt strains because of an insertion of A in position 861; most ∆*ggt* isolates without the insertion | insertion of A leads to a frameshift and disruption of the gene;  full length protein is restored in most ∆*ggt* isolates |
| HPYL  PMSS1_  01343 | *cptA* | Phosphoethanolamine  transferase | A0A3Q9Y238 | wt BI: full length gene ∆*ggt* BI: GA:del at position 805, 806 in 1 W ∆*ggt* control wt RIs: full length gene  ∆*ggt* RIs: full length gene, GA:ins in position 807 in one of the RIs | only full length protein in RIs; disrupted gene in some ∆*ggt* BI clones |
| HPYL  PMSS1_  01449 |  | Site-specific  DNA-methyltransferase  (adenine-specific) | A0A3T1CRE4 | G stretch with varying amounts of G at the end of the gene;  more G in wt isolates | depending on amount of G a frameshift leads to the disruption of the gene;  gene is disrupted in all wt RIs and in 3/6 ∆*ggt* RIs |
| HPYL  PMSS1_  01465 | *fecA3* | Fe(3+) dicitrate  transport protein | A0A3T1CVF8 | A stretch in the promoter region with varying amounts of A;  more A in wt isolates |  |
| HPYL  PMSS1_  01469 |  | DUF262 containing protein | A0A3Q9Y098 | varying number of A in an A-stretch in the promoter region;  more A in wt isolates |  |

**Grey: not detected as difference by proteome analysis; RI: re-isolate, BI: before infection, del: deletion, ins: insertion**

**Table S9: Overview of genomes sequenced in this work**

| **genotype** | **time point** | **before infection /after infection** | **name in sequencing data** |
| --- | --- | --- | --- |
| PMSS1 wt | 1 week | before infection | 1 W wt control |
| PMSS1 wt | 1 week | after infection, re-isolate | M1 |
| PMSS1 wt | 1 week | after infection, re-isolate | M2 |
| PMSS1 wt | 1 week | after infection, re-isolate | M3 |
| PMSS1 ∆*ggt* | 1 week | before infection | 1 W ggt control |
| PMSS1 ∆*ggt* | 1 week | after infection, re-isolate | M5 |
| PMSS1 ∆*ggt* | 1 week | after infection, re-isolate | M6 |
| PMSS1 ∆*ggt* | 1 week | after infection, re-isolate | M11 |
| PMSS1 wt | 1 month | before infection | 1 M wt control |
| PMSS1 wt | 1 month | after infection, re-isolate | M16 |
| PMSS1 wt | 1 month | after infection, re-isolate | M13 |
| PMSS1 ∆*ggt* | 1 month | before infection | 1 M ggt control |
| PMSS1 ∆*ggt* | 1 month | after infection, re-isolate | M19 |
| PMSS1 ∆*ggt* | 1 month | after infection, re-isolate | M20 |
| PMSS1 ∆*ggt* | 1 month | after infection, re-isolate | M23 |
| PMSS1 wt | 3 months | before infection | 3 M wt control |
| PMSS1 wt | 3 months | after infection, re-isolate | M25 |
| PMSS1 wt | 3 months | after infection, re-isolate | M27 |
| PMSS1 wt | 3 months | after infection, re-isolate | M28 |
| PMSS1 ∆*ggt* | 3 months | before infection | 3 M ggt control |
| PMSS1 ∆*ggt* | 3 months | after infection, re-isolate | M31 |
| PMSS1 ∆*ggt* | 3 months | after infection, re-isolate | M29 |
| PMSS1 ∆*ggt* | 3 months | after infection, re-isolate | M36 |

**Supplementary Figures**


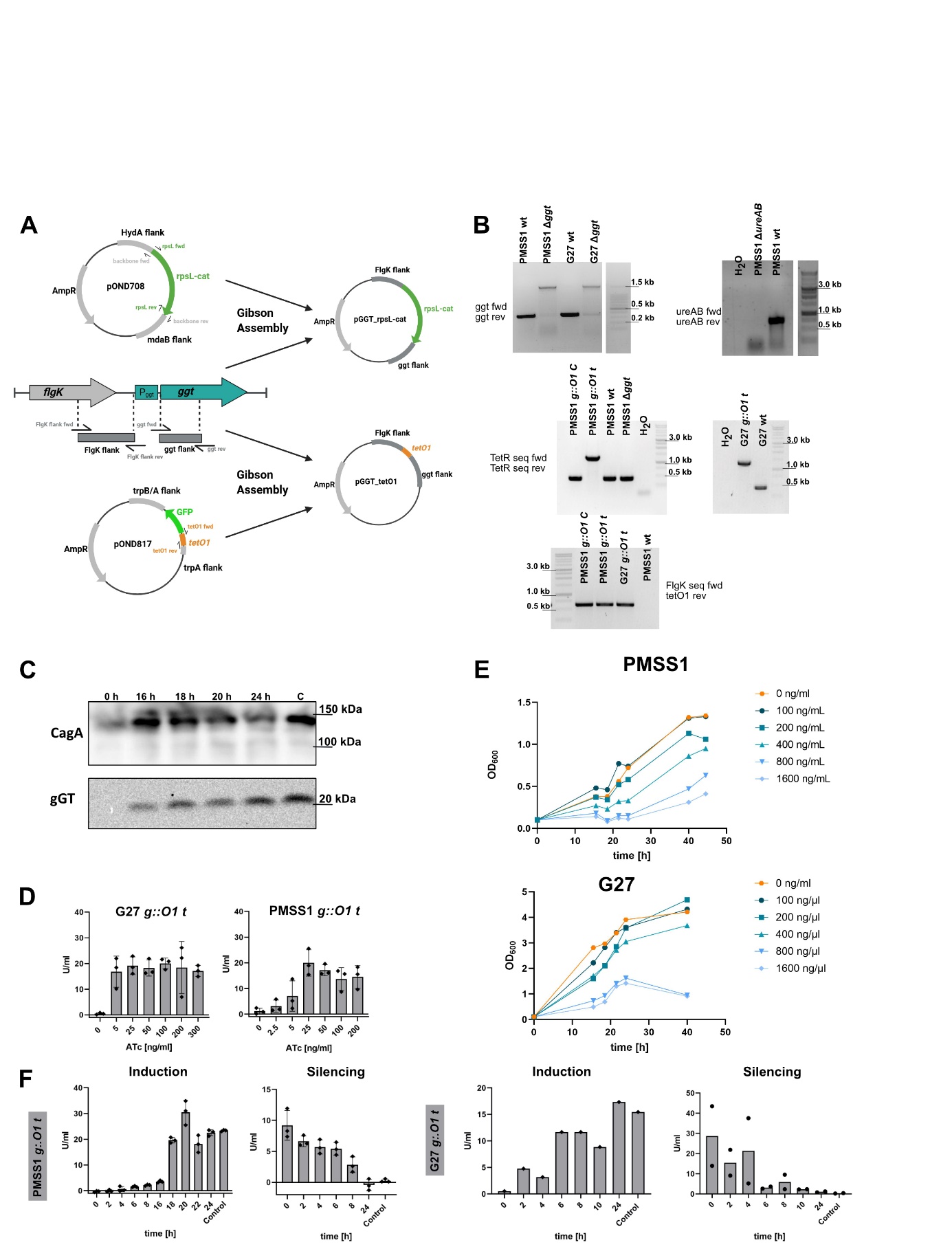


**Figure S1: Establishment and characterization of inducible tet-on gGT H. pylori strains**

(**A**) Schematic representation of the cloning procedure. (**B**) PCR confirmation of mutant strains used in this study. Primer pairs ggt fwd/rev, ureAB fwd/rev, TetR seq fwd/rev and FlgK seq fwd/tetO1 rev were used to detect the presence of *ggt,* *ureAB*, *tetR* and *tetO1* in the *H. pylori* genome. (**C**) Western Blot detecting *tetR*-controlled induction of gGT-expression in PMSS1 *g::O1 t.* For induction, 100 ng/ml ATc was added to the culture. As a control lysate of PMSS1 *g::O1 t* after induction for 24 h was used. Equal amounts of protein (2 µg) were loaded in each lane. (**D**) gGT-activity in the presence of different ATc concentrations after 24 h of culture in BHI/10% FCS. The same amount of solvent (ethanol) was added to all cultures as a control, n= 3. (**E**) Growth of *H. pylori* wt in the presence of different ATc concentrations. Growth was monitored spectrophotometrically at 600 nm. ATc was dissolved in ethanol. As solvent control the same amount of ethanol was added to each culture. Shown is one representative experiment per strain. n=3 (**F**) TetR controlled induction and silencing of gGT-activity in PMSS1 *g::O1 t* or G27 *g::O1 t*. For induction, 100 ng/ml ATc was added to the culture (starting concentration: 1x10^8^ bacteria/ml). As a control the gGT-activity after induction for 24 h was measured. For silencing, gGT-activity was induced for 24 h with 100 ng/ml ATc during growth in BHI/10% FCS. The culture was washed with PBS and resuspended in ATc-free medium at a concentration of 1*10^8^ bacteria/ml. As a control, an uninduced 24 h old culture of G27 *g::O1 t* was used, n=1-3. Bars represent the mean of one to three independent experiments as represented by single dots. n: number of independent experiments.


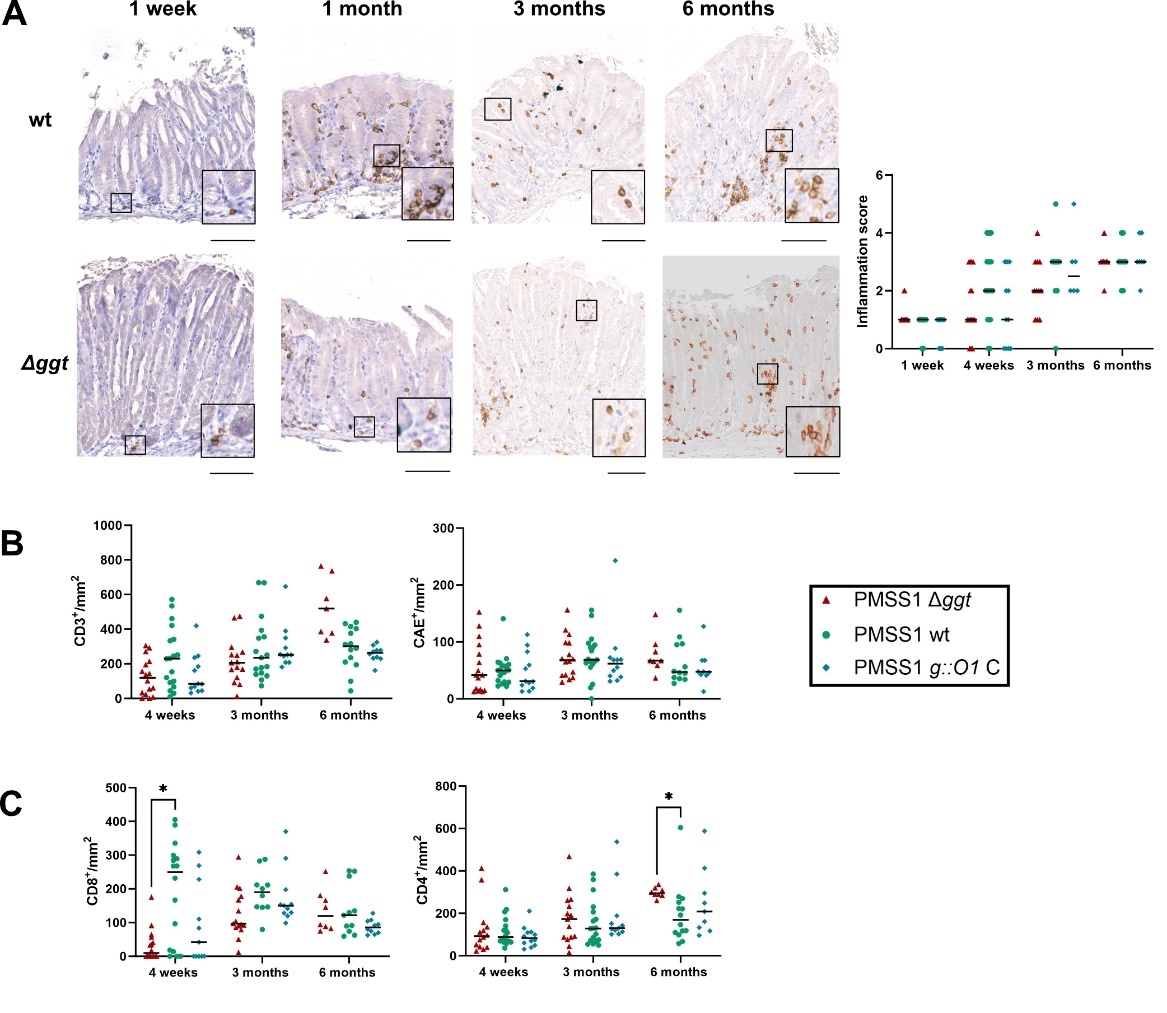
Determination of immune cells in the murine stomach of mice infected with PMSS1 wt, ∆*ggt* or *g::O1 C* after 1 week, 1 month, 3 months or 6 months of infection. Each dot represents one individual mouse. (**A**) Gastric inflammation in murine stomach sections. Representative CD3 immune histochemistry stainings are shown. Inflammation was scored based on the updated Sydney classification.^11^ Infiltration of CD3^+^ and chloroacetate esterase (CAE^+^) positive cells was graded as normal (score 0), mild (1), moderate (2) or marked (3). These grades were combined to an inflammation score on a scale of 0-6. (**B**) CD3 and CAE stainings of murine stomach sections. CD3^+^ and CAE^+^ per mm^2^ were counted. (**C**) CD4 and CD8 stainings of murine stomach sections. CD4^+^ and CD8^+^ per mm^2^ were counted.. Multiple Mann-Whitney U-tests followed by Dunn’s test for multiple comparisons. *** p<0.05.

**Figure S2: Immune cell infiltration into the stomach of H. pylori infected mice**


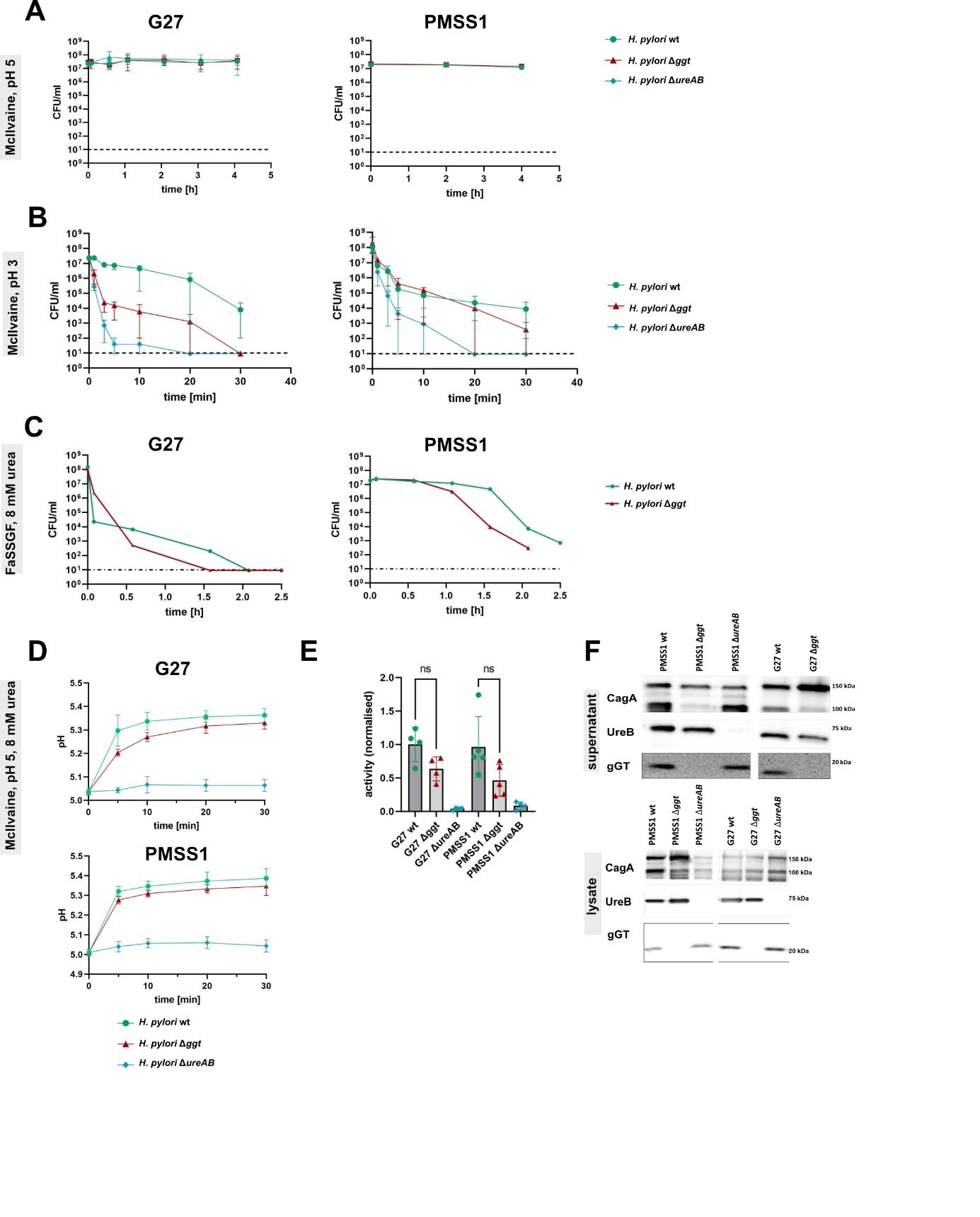


**Figure S3: *H. pylori* gGT contributes to acid survival**

(**A, B and C**) Survival of H. pylori in McIlvaine buffer without supplementation at pH 5 (A) or pH 3 (B) and in fasted state simulated gastric fluid (FaSSGF) (C), viable cell counts were monitored by plating. Shown are the means and range of three independent experiments (A and B) or one representative experiment (n=3) (C). The detection limit of the assay is indicated by a dotted line. (**D**) H. pylori induced urea-dependent pH increase. Bacteria were grown on WC-dent plates and resuspended in McIlvaine buffer (4*10^8^ bacteria/ml) pH 5 with 8 mM urea. PH was monitored with a handheld pH electrode. (**E**) Urease activity in the supernatant after exposure of H. pylori for 2 h to pH 5. Strains were grown in BHI with 10% FCS for 24 h and resuspended for 2 h in McIlvaine buffer (pH 5) before measurement. Data was normalized to the mean activity of H. pylori wt. Bars represent the mean of 4-5 independent experiments as indicated by single dots. Error bars represent the standard deviation. (**F**) Western blot detection of urease subunit B in the supernatant and lysate. The same amounts of protein were loaded (supernatant: 0.5 µg, lysate: 5 µg). CagA was used as a control. n: number of independent experiments. ANOVA (D, E).


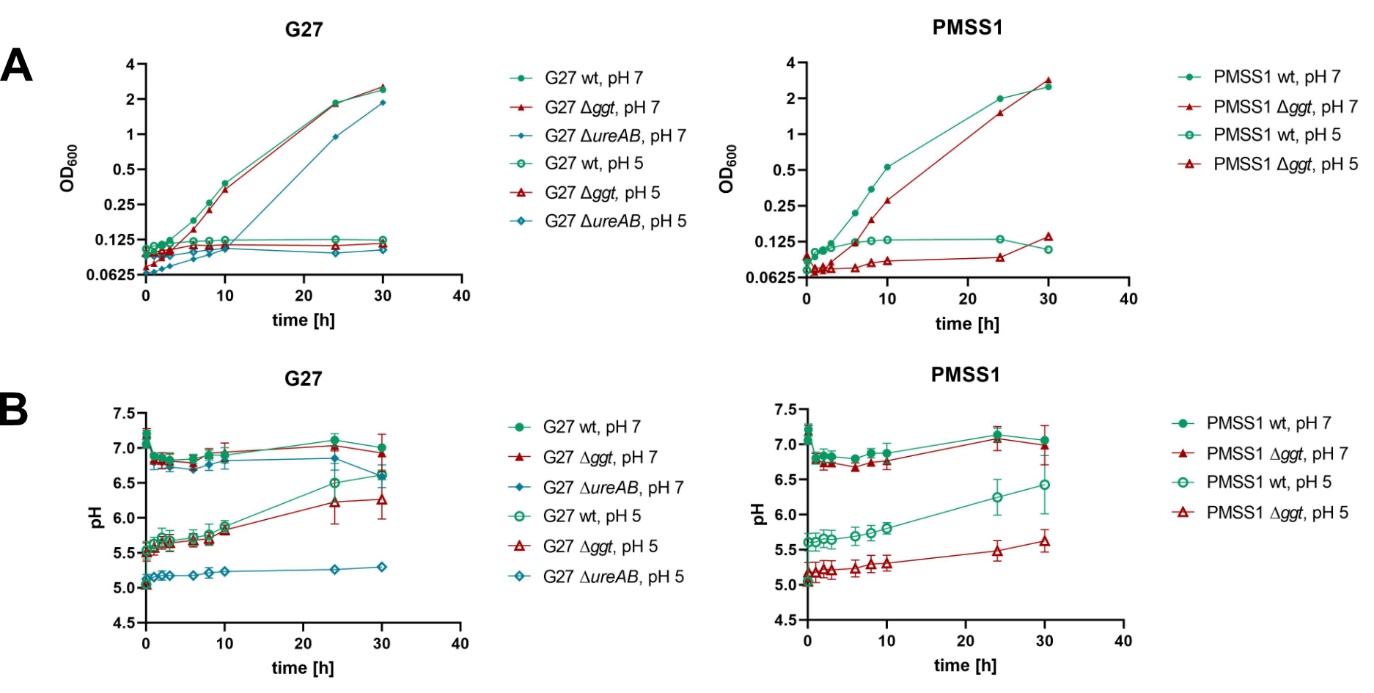


**Figure S4: Growth and pH increase of H. pylori at low pH**

(**A**) Growth of H. pylori in BHI medium with 20% FCS at pH 5 and pH 7. Growth was measured spectrophotometrically at 600 nm (OD_600_). One representative experiment is shown. (n=2) (**B**) pH increase during growth in Brucella medium supplemented with 20% FCS at pH 5 and pH 7. PH was measured with a handheld electrode. (n=3). n: number of independent experiments.


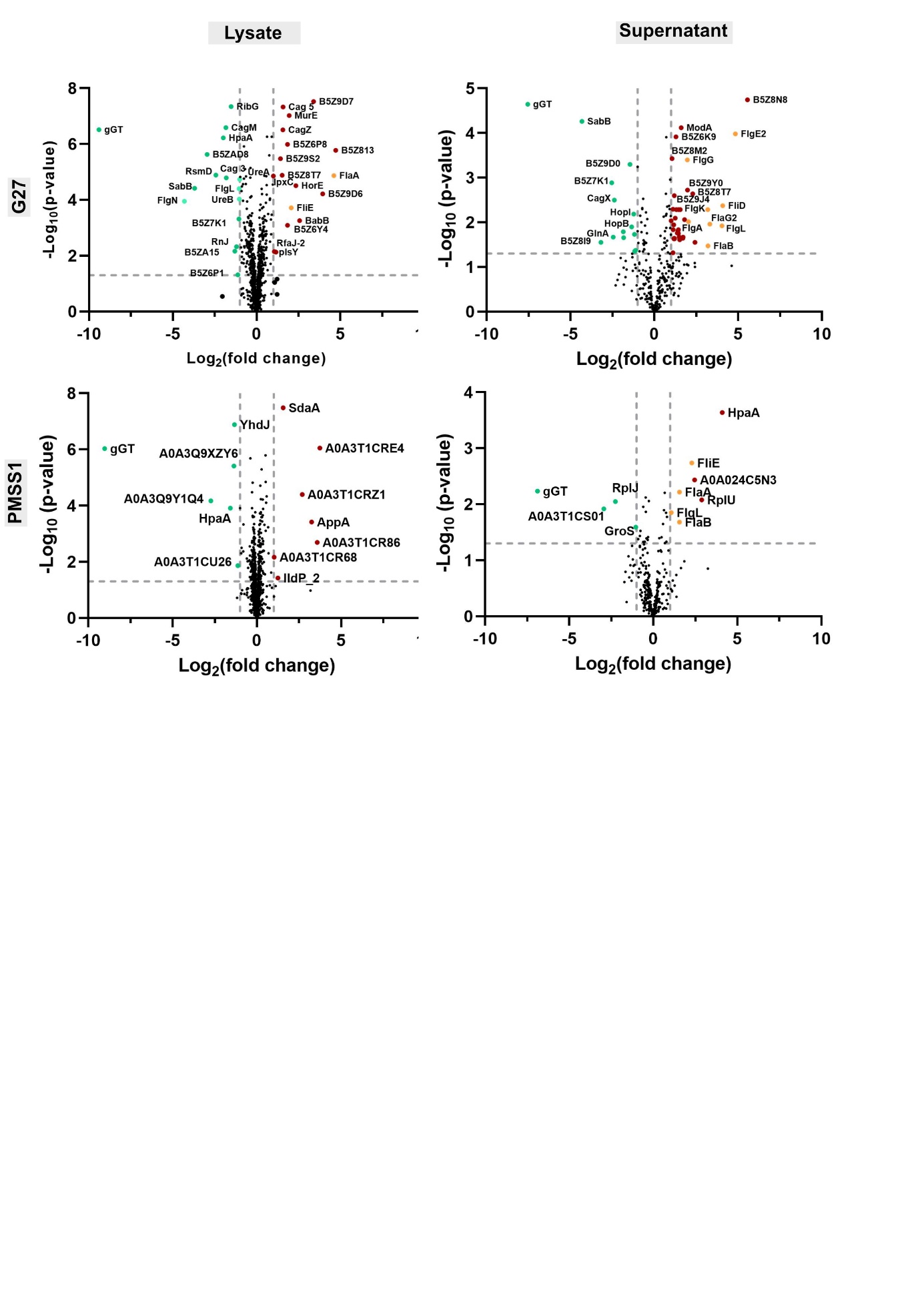


**Figure S5: Comparison of the proteome and secretome of *H. pylori* wt and ∆*ggt* after exposure to pH 5**.

The proteomes and secretomes of *H. pylori* wt and ∆*ggt* were determined in triplicates. Thresholds for significance (-log_10_(p-value) > 1.3 and log_2_(fold change) ≥ I1I) are represented by dotted lines. Proteins stronger expressed by the wt are marked in green, proteins stronger expressed by the gGT-deficient mutants are marked in red. Flagellar proteins are highlighted in orange. Students t-test.


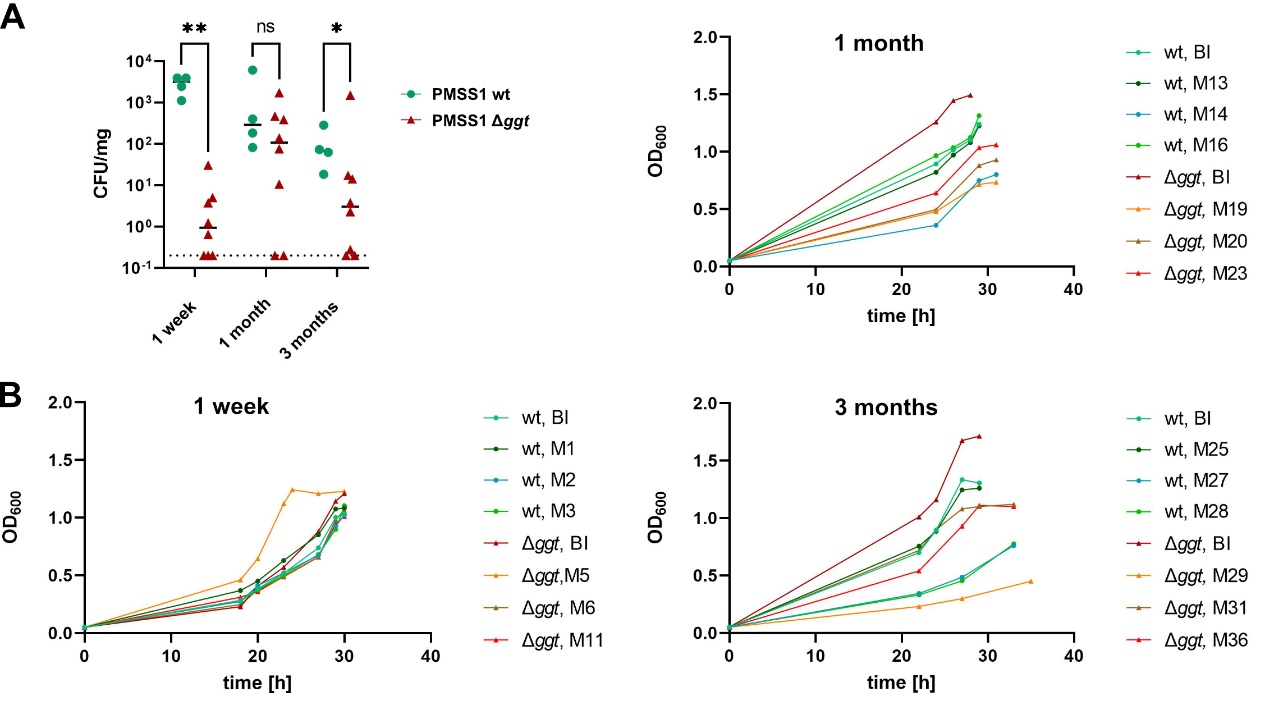
**Figure S6: Preparation for proteome comparison of *H. pylori* isolates**

(**A**) Infection level of mice used for re-isolation of *H. pylori*. C57Bl6 mice were inoculated twice with 2*10^8^ bacteria at a time interval of 2 days. Mice were sacrificed after 1 week, 1 month of 3 months of infection and colonization level was assessed by plating stomach homogenates. (**B**) Growth curves of re-isolates and strains initially used for infection (BI). Isolates were picked, expanded for 2 d on WC-dent plates and cultivated in BHI/10% FCS for a maximum of 35 h or until the stationary growth phase was reached. Mann Whitney U-test. *p < 0.05, **p < 0.01


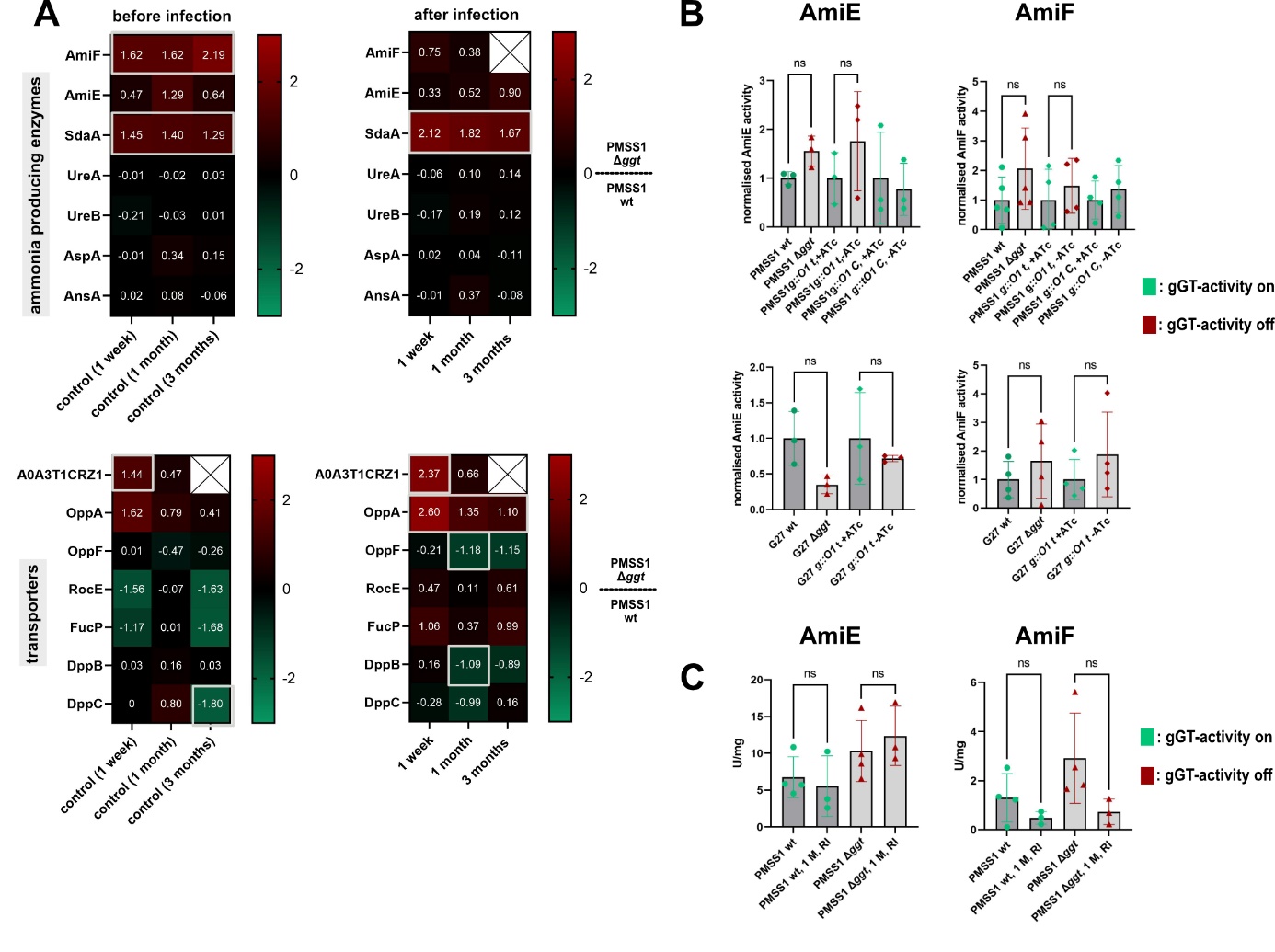
 **Figure S7: Proteins involved in nitrogen metabolism are differentially expressed in the absence of gGT**

**(A**) Log_2_ transformed fold change of ammonium producing enzymes and of peptide/metabolite transporters comparing the proteome of PMSS1 wt and ∆*ggt*. The proteomes of three single clones isolated after 1 week, 1 month and 3 months of infection from different mice were determined. Significant differences (-log_10_(p-value) > 1.3 and log_2_(fold change) ≥ I1I) are marked with grey boxes. Missing data points are colored white and are marked with a cross. (**B**) AmiE and AmiF activity of *H. pylori* normalized to the mean activity of *H. pylori* wt or tet-on *H. pylori g::O1 t* (**C**) AmiE and AmiF activity of *H. pylori* strains before infection (BI) and of re-isolates (RI). Strains were grown for 24 h in BHI/10% FCS before measurement. RI were retrieved from mice stomachs after 1 month of infection, expanded for 2 d on plates and frozen for further use. Bars represent the mean of three to four independent experiments as indicated by dots. Error bars represent the standard deviation. Students t-test (A). ANOVA (B,C). *** p<0.05.


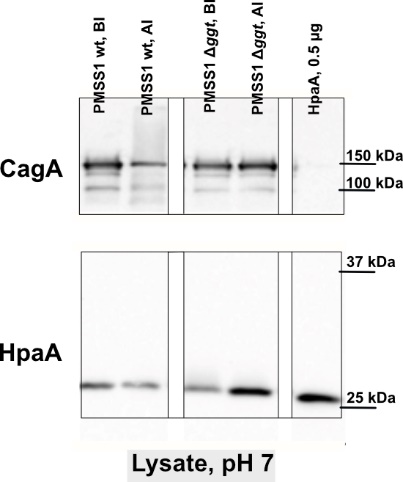
**Figure S8: Immunoblot detection of HpaA**

Bacteria were grown for two days on WC DENT plates and were lysed with RIPA buffer. 5 µg lysate and 0.5 µg recombinant HpaA were loaded on the SDS gel. CagA was used as a control and recombinant HpaA as a positive control.

**Supplementary references**

1. Dailidiene D, Dailide G, Kersulyte D, Berg DE. Contraselectable streptomycin susceptibility determinant for genetic manipulation and analysis of helicobacter pylori. Appl Environ Microbiol. 2006; 72(9): 5908-5914. doi:10.1128/AEM.01135-06

2. Debowski AW, Walton SM, Chua EG, Tay AC, Liao T, Lamichhane B, Himbeck R, Stubbs KA, Marshall BJ, Fulurija A, Benghezal M. Helicobacter pylori gene silencing in vivo demonstrates urease is essential for chronic infection. PLoS Pathog. 2017; 13(6): e1006464. doi:10.1371/journal.ppat.1006464

3. Debowski AW, Verbrugghe P, Sehnal M, Marshall BJ, Benghezal M. Development of a tetracycline-inducible gene expression system for the study of helicobacter pylori pathogenesis. Appl Environ Microbiol. 2013; 79(23): 7351-7359. doi:10.1128/AEM.02701-13

4. Wisniewski JR, Zougman A, Nagaraj N, Mann M. Universal sample preparation method for proteome analysis. Nat Methods. 2009; 6(5): 359-362. doi:10.1038/nmeth.1322

5. Skouloubris S, Labigne A, De Reuse H. Identification and characterization of an aliphatic amidase in helicobacter pylori. Mol Microbiol. 1997; 25(5): 989-998. doi:10.1111/j.1365-2958.1997.mmi536.x

6. Xiang Z, Censini S, Bayeli PF, Telford JL, Figura N, Rappuoli R, Covacci A. Analysis of expression of caga and vaca virulence factors in 43 strains of helicobacter pylori reveals that clinical isolates can be divided into two major types and that caga is not necessary for expression of the vacuolating cytotoxin. Infection and Immunity. 1995; 63(1): 94-98. doi:doi:10.1128/iai.63.1.94-98.1995

7. Arnold IC, Dehzad N, Reuter S, Martin H, Becher B, Taube C, Muller A. Helicobacter pylori infection prevents allergic asthma in mouse models through the induction of regulatory t cells. J Clin Invest. 2011; 121(8): 3088-3093. doi:10.1172/JCI45041

8. Schmees C, Prinz C, Treptau T, Rad R, Hengst L, Voland P, Bauer S, Brenner L, Schmid RM, Gerhard M. Inhibition of t-cell proliferation by helicobacter pylori gamma-glutamyl transpeptidase. Gastroenterology. 2007; 132(5): 1820-1833. doi:10.1053/j.gastro.2007.02.031

9. Oertli M, Noben M, Engler DB, Semper RP, Reuter S, Maxeiner J, Gerhard M, Taube C, Muller A. Helicobacter pylori gamma-glutamyl transpeptidase and vacuolating cytotoxin promote gastric persistence and immune tolerance. Proc Natl Acad Sci U S A. 2013; 110(8): 3047-3052. doi:10.1073/pnas.1211248110

10. Kaebisch R, Mejias-Luque R, Prinz C, Gerhard M. Helicobacter pylori cytotoxin-associated gene a impairs human dendritic cell maturation and function through il-10-mediated activation of stat3. J Immunol. 2014; 192(1): 316-323. doi:10.4049/jimmunol.1302476

11. Dixon MF, Genta RM, Yardley JH, Correa P, the Participants in the International Workshop on the Histopathology of Gastritis H. Classification and grading of gastritis: The updated sydney system. The American Journal of Surgical Pathology. 1996; 20(10): 1161-1181.
